# Thermal buffering with medium-temperature PCM for enhanced solar still performance in hot Egyptian conditions

**DOI:** 10.1038/s41598-026-47006-7

**Published:** 2026-04-18

**Authors:** M. Elsayed, M. Salah Mansour, H. Yahya, M. A. Eid, Moataz. M. Abdel-Raouf

**Affiliations:** 1 Mechanical Engineering Dept, Higher Technological Institute, 10th of Ramadan City , 44629 Egypt; 2 Mechanical Engineering Dept, Faculty of Engineering, City University of Cairo, CUC, Cairo, Egypt

**Keywords:** Carbon mitigation, Energy and exergy analysis, Hot domestic water coupling, Passive Solar still, Phase change material (PCM), Renewable energy desalination, Thermal storage hybrid systems, Climate sciences, Energy science and technology, Engineering, Environmental sciences

## Abstract

Solar-still desalination is a promising, sustainable option for decentralizing freshwater production in areas with useful solar irradiation. Nevertheless, the relatively low productivity of conventional solar stills is always an obstacle to their large-scale application. In response to this challenge, thermal energy storage has been a subject of extensive research as a potent method that can be employed in the solar desalination system via phase-change materials (PCMs). The current study integrated a medium-temperature PCM into a single-slope solar still to enhance thermal energy storage and prolong the effective evaporation period. Moreover, an experimental investigation of the hot domestic water “HDW heat exchange”-assisted hybrid configuration was performed to further improve the savings of stored thermal energy. Three identical solar still designs were investigated under the same outdoor conditions, specifically a conventional solar still (CSS), a PCM-assisted solar still (PCM-SS), and finally a hybrid solar still with PCM storage and HDW heating hybrid solar still (HSS). Experimental findings during the evaluated summer revealed that the implementation of PCM accordingly increased freshwater production over conventional solar stills. Under the experimental conditions, the average daily distillate yield was 1.428 L/day for CSS and 2.482 L/day for the PCM-assisted system, representing an improvement of about 73.8%. The maximum daily energy efficiency was 24.77%, and the exergy efficiency was 7.10%. From an economic viewpoint, the PCM-assisted configuration obtained a lower cost per liter of produced freshwater than the conventional design under the adopted economic assumptions. The environmental assessment also suggested a possible decrease in carbon dioxide emissions linked to freshwater production. Because the current experiments were performed over a limited summer measurement period, long-term economic and environmental indicators presented in this study should be viewed as scenario-based estimates rather than direct year-round measurements. More studies are suggested to confirm system behavior in other seasons and explore the PCM’s long-term performance under different climate conditions.

## Introduction

With more than 2.2 billion people worldwide lacking access to clean drinking water and 4.2 billion lacking adequate sanitation services, the global water crisis is one of humanity’s greatest threats. By 2050, the world’s population is expected to exceed 9.7 billion creating a 55% increase in demand on freshwater supplies. Climate change is altering precipitation patterns, increasing the frequency of droughts, and accelerating glacier retreat. Sustainable, high-tech water treatment methods are needed to combat this crisis. The abundance of solar energy together with growing water security requirements provides a promising opportunity for solar desalination technologies. Solar desalination, which draws on solar radiation and needs little infrastructure, contrasts with energy-intensive processes such as reverse osmosis (3–4 kWh of electricity per cubic meter of freshwater), multi-stage flash distillation or thermal vapor compression. This fundamental sustainability advantage is what makes the system particularly desirable in remote communities and underserved areas, where access to grid electricity is costly and unpredictable. The productivity of solar desalination is low in common practice: 2 to 5 L/m2 per day, even under optimal conditions. This productivity problem has already sparked huge interdisciplinary research efforts in areas such as materials science, thermal science and engineering, nanotechnology, and system optimization. Empowering working with PCM, NCA, and hybrid arrangements alone has produced over 200% improvement in the overall efficiency compared to the traditional solar stills^1^.

Other studies in recent years have concentrated on the coupling of solar still systems with other external thermal energy storage units and solar concentrators to increase operational hours and enhance total productivity. Recent experimental studies have shown that thermal efficiency and freshwater generation can be markedly enhanced through the combination of solar stills with external storage components and combined Fresnel lens concentrators under variable salinity levels^2^. The potential for transforming solar desalination by phase change materials (PCMs) is substantial. The combination of various phase change materials available has also been studied recently to maximize heat storage and improve desalination. This combination of dual PCM materials has been shown to enhance freshwater outputs and energy and exergy efficiencies and decrease production costs in comparison with traditional solar still systems^3^.

Previous studies have also examined several organic phase change materials to increase the performance of solar stills. For example, Dhivagar et al. reported that, as the PCM thermal storage media, the inclusion of beeswax and paraffin wax could contribute to solar still systems becoming more productive in terms of freshwater output while also showing improved energy efficiency and economic feasibility when compared unit for unit under identical operating conditions^4^.

The stored thermal energy can be used in the low solar radiation period to keep generating electricity during the day by converting it to electrical energy using a heat engine, which operates only during daylight hours, reconciling the natural intermittency of solar energy^1,5,6^. There are some studies that have reported that nano-enhanced PCM can increase the thermal performance by 71%^1^. Under the same conditions as other materials, such as RT70 HC, special performance has been achieved, with cumulative output increasing from 4.53 to 5.42 kg/m² and total efficiency rising from 55.78% to 66.70%^5^. Studies have been carried out on the performance of fin-shaped absorbers in combination with paraffin wax PCMs, achieving a maximum daily output rate value of 5.62 L, which implies a productivity improvement of 87.96%; see Ref^7^. One such step forward is the advanced PCM-integrated systems with graphite, which have a heat transfer coefficient that is 221.8% higher than that of typical tubular solar stills^8^.

Also touched upon are the extraordinary opportunities for performance enhancements based on the tailored properties of materials, which have been opened up by simultaneous progress in nanotechnology. The CuO/GO nanocomposite incorporation offers 81.59% high yield of freshwater production, and the optimal volume ratio matrix (30/70) has shown better performance while maintaining distilled water quality. These nanocomposites with enhanced properties can combine the excellent thermal conductivity, better light absorption, and heat-carrying capacities, which are expected to lead to multiplicative performance enhancements rather than just adding up benefits^9^. In the project of solar desalination, thermoelectric material can play a very comprehensive role: a cooler for increasing the efficiency of the condenser, a heater for enhancing evaporation, and a generator for transforming thermal energy to electricity for a hybrid system running^10^. The trend toward hybrid and integrated systems is an indication of the future evolution that SD technology will undergo.

Recent interest is also focused on solar stills integrated with solar concentrators and heat-sensible storage materials. By the use of experimental investigations performed by integrating parabolic trough concentrators with stainless-steel thermal storage elements, local productivity of freshwater, thermal efficiency, and environmental performance are enhanced when compared to conventional solar still configurations^11^. Similar to other recent research, novel thermal storage and heat-transfer enhancement approaches have been investigated in the context of increased solar still productivity. For example, recent studies on coupled conical solar distillers with copper cone fins filled with phosphate composite materials provide integrated designs that attract enhanced heat transfer in addition to sensible thermal storage while maintaining optimized freshwater productivity and overall efficiency due to the interaction of these effects. The proposed arrangement achieved a maximum yield of 8.2 L m⁻² day⁻¹, compared with the traditional conventional solar still, which yielded around 4.8 L m⁻² day⁻¹, indicating a productivity increase of approximately 69.8%. Furthermore, the hybrid system demonstrated superior thermal efficiency as well as greater potential for CO₂ removal performance, underscoring this the effectiveness of integrating high-performance materials with solar desalination systems. These results strongly confirm the need for employing thermally energy storage mechanisms to mitigate the intermittence of solar radiation, resulting in a longer operational time for a solar still system^12^.

The PV-integrated systems are 43% more effective than the existing ones^13^, and in the multistage hybrids comparable to our case, several enhancements were established with synergistically enhanced performance beyond that of individual components^14^. Other studies have also explored the hybridization of photovoltaic panels with solar still systems coupled with phase change material to synergistically improve energy utilization and freshwater yield. Experimental results showed that integrating a solar still with a coupled SPV panel and A46 phase-change material enhances the performance of the system nearly 245% higher than conventional double-slope solar stills in terms of productivity, as well as higher economic feasibility than conventional systems and lower operating costs. Such photovoltaic–thermal hybrid desalination systems represent an increasing potential of hybrid renewable energy configurations in enhancing the sustainability and economy of solar desalination technologies^15^. Innovative evaporative coolers employing natural fiber-based materials such as sisal fiber arrangements are reported to result in productivity improvements of 104.5% on standard solar stills and 19.1% more than conventional glass cooling systems^16^. The use of flat plate collectors greatly enhances the utilization of proximate solar energy, leading to 41% and 89% improvements for the two- and three-connected collector configurations, respectively^17^. For example, finned solar still designs with nanoparticle-enhanced heaters and phase change materials provide the improvements of 166% and 136% in production obtained relative to that of conventional CSS systems^18^. The share of passive mode is roughly 39.82% and 2.9% for thermal and exergy efficiency, respectively, which is acceptable in the ISPB case. The active mode systems enhanced the production of fresh water by 46.87% and 6.6%^19^. The advantages of the graphite storage boost are substantial from a performance perspective, as the graphite-enhanced efficiencies fall in the range of 59.9–60.54% vs. that of a standard system at 33.41–34.6%^20^. Economic feasibility analysis with a complete 4E (Energy, Exergy, Economic, and Environment) assessment framework was carried out, which reveals that an optimally designed adsorption solar still system gives an average energy efficiency of 26.51%, an exergy efficiency of 2.58%, an economically viable production cost of $0.0084 per liter, and the potential significant carbon credit earnings of $183.5^21^. In low-populated areas (less than 10,000), the corresponding small-scale photovoltaic-driven desalination systems provide fresh water at a cost of less than $7.71/cu.m, showing that they are competitive with conventional technologies and offer great potential in achieving energy independence and meeting environmental sustainability. The investigations of a mathematical model using optimization techniques in MATLAB show that a solar still coupled with PCM produces 4.5 L/m²/day at the cost of INR 42.34/m³ (€4.31), and without PCM, it yields a production of 4.1 L/m²/day, and the cost is $43.60 per m³, which shows CCDRM is an eco-friendly technology^22^. Regional optimization investigations also have shown that there are radical performance enhancements achievable due to climate-specific changes: Middle Eastern applications of reticular porous layer enhancement were found to be capable of gaining a 17.35% increase in productivity (3,829 cc/m² compared with 3,263 cc/m² using traditional systems)^23^. In a triangular basin, the meteorological temperatures are 6.8% higher than those in square basins; triangular basins collect an energy value of 3.67 kg/m² daily, compared to 3.29 kg/m² from square basins^24^. For low-cost increases in production and efficiency, alternative thermal storage materials such as black gravel provide cost-effective enhancement options that can lead to production gains of 10–17% and efficiency improvements of 12–16%^25^. Porous absorber systems, on the other hand, had a considerable improvement in heat and mass transfer, being about 115%, 51.95%, and 123%, as compared to ordinary solar stills, with exergy actually up by an increase of 65%, 1.5%, and 104%^26^. Research using a different approach with alternate sources of energy on optimization strategy for energy storage reveals complex relationships between thermal mass and system performance, with a solar still devoid of mass reaching 35.1% efficiency, but systems that use stone for storing energy achieve 29.3% efficiency^6^. Composites with internal reflectors and black gravels provide an average thermal capacity of 3.27 L/m² that is produced (i.e., a 37.55% improvement over the baseline configuration), whereas the combination of PCMs yields a 38% increase in energy efficiency and a 37% increase in exergy efficiency^27^. From the monthly performance simulation, it is concluded that annual yield strongly depends on water depth and collector geometry parameters^28^. The ideal design of the hydraulic system should consider fluid velocity, flow rate, basin depth, and environmental conditions^29^. Studies on the potential and optimal flow rates show that an optimum rate of 4.56 kg/hr was observed, whereas higher rates of 7.56 kg/hr and 10.08 kg/hr caused a daily yield reduction of about 27% and 57%, respectively^30^.

Although considerable progress has been made in the performance of solar power over recent decades, most such previous works have focused on optimizing the contribution of a parameter rather than pursuing integrated optimization methods. These works mainly concern the improvements of latent heat storage systems with phase change materials (PCM)^31^. While others have individually assessed several other enhancement methods for thermal energy storage systems, including geometric modifications and nanomaterial loading techniques, as well as solar concentrators or assistance heating appliances. Therefore, the interaction and synergistic effects of multiple enhancement strategies under realistic environmental conditions remain poorly characterized. Recent studies on hybrid solar desalination systems linked to thermodynamic analyses have shown that the combination of various enhancement mechanisms can bring about significant improvements in fresh water productivity and overall sustainability for mains desalination systems and low-cost installations alike^32^.

Despite all this progress, though, there are several really big challenges that remain. These include identifying optimal combinations of materials and system configurations; ensuring for long-term operating durability under broad climatic conditions; and developing economically viable and scalable systems and scalable considerations for systems implementations. Moreover, very little literature includes both thermodynamic performance and economic feasibility or environmental impact assessment together at this moment. Therefore, additional experimental studies are needed that simultaneously compare thermal energy storage materials and hybridization strategies in hybrid heating configurations, as well as elucidate the relative impacts of these hybridization configurations on solar desalination systems’ performance under realistic operating conditions from a thermodynamic, economic, and environmental perspective. The present work intends to further clarify the relationships between material innovation and system design optimization, as well as economic feasibility versus practical implementation issues. In so doing, it aims to help pave the way towards filling the space between laboratory-based demonstrations and large-scale implementation of sustainable solar desalination technologies^1,6,8,10,20,27,29^. The results of this work are anticipated to researchers, engineers, and policymakers who are working toward developing cost-effective water desalination technologies directed at sustainably enabling global water security.

### Contribution of the current study

Solar stills represent a promising technology for water purification that converts brackish and seawater into potable water, making them very suitable for faraway regions and small towns, among which standard facilities are impossible. However, due to the very low performance efficiencies of conventional solar stills, a significant amount of work has been done on enhancement techniques and parametric optimization to improve their productivity. In this work, an attempt is made to address several research gaps identified in the field by investigating the integration of medium-temperature PCMs for thermal energy storage (TES), including salt hydrates and paraffin, on enhancing daily yield from single-slope solar stills. This study has several specific contributions:

- Comprehensive 4E analysis for solar desalination in the Middle East: This study provides a comprehensive cross-disciplinary evaluation and integrated evaluation of an Energy, Exergy, Economic, and Environmental (4E) approach for PCM-influenced solar stills working in a marine region in the Middle East, having critical regional Performance indicators which could be supportive before technology deployment.
- Thermodynamic optimization of PCM mass: Seeking the best thermal storage configuration by an experimental approach with diverse PCM masses in contrast to theoretical prediction.
- Medium-Temperature PCM performance investigation: Shortly describe the SP 48 PCM performance characteristics at realistic operating conditions, which adds to a scarce database on medium-temperature PCMs used in solar desalination.
- Coupled thermodynamic and economic evaluation: The full study correlates the same improvements in thermal operation with a cost evaluation comprehensive enough to be useful for practical commercial deployment within the Middle East.

## Experimental setup

### The purpose and arrangement of this research

This experimental investigation is intended to evaluate the performance improvement that can be achieved with PCM storage modules incorporated into solar still systems. The work contains energy saving, exergy calculation, cost estimation, environmental effect, and freshwater production.

**Fig. 1:**
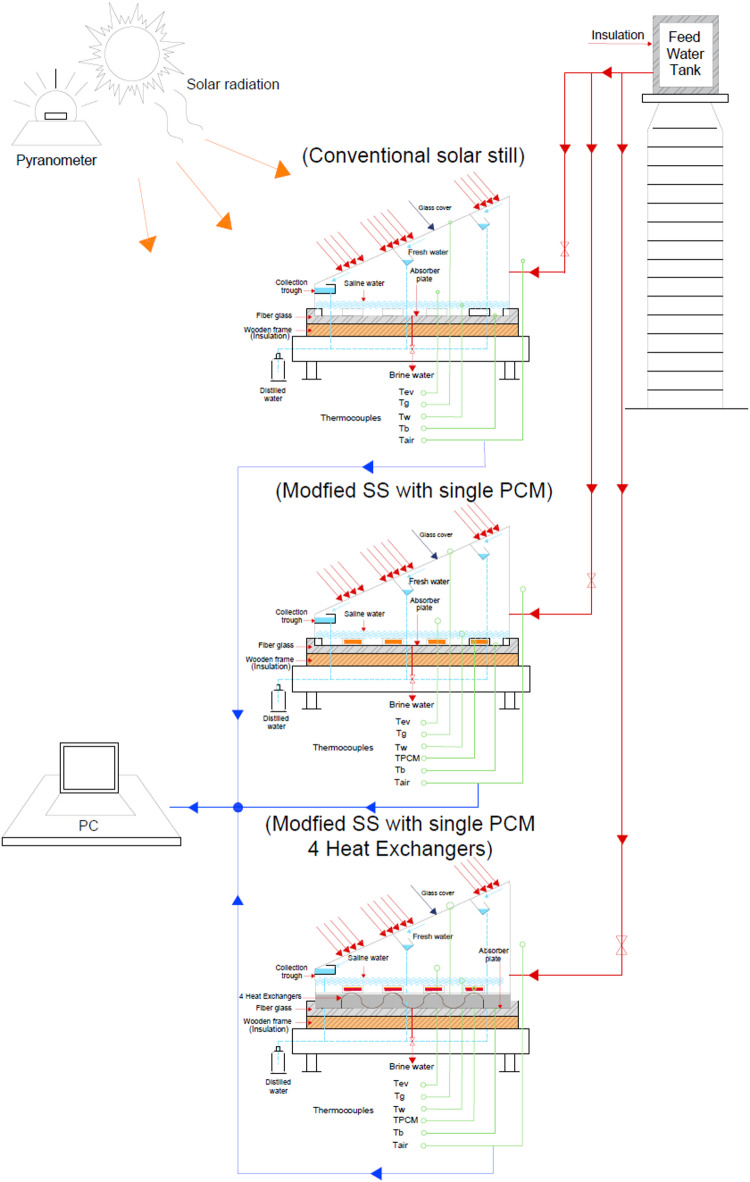
Schematic of the test rig.

The experimental configuration includes a set of three identically paired single-slope PSCS stills produced using similar performance specifications and the same material. These models were tested before PCM modifications and demonstrated stable performance of the base case before modification. This cumulative process provides a solid common justification for the performance comparison of PCM-integrated cases against the reference integral regular case. Figure 1 shows the complete schematic of the test rig while providing information about the structural arrangement and geometric distribution for all three solar still systems considered in this comparison study. The solar stills were all perfectly inclined in a southern direction on the east-west axis in order to be able to capture as much direct solar radiation during daytime hours as possible. By applying the angle of the condensing glass to be equal to that day’s latitude angle, maximum absorbed incident solar radiation is achieved. To allow the environmental parameters to remain similar throughout all test scenarios, all experimental investigations were performed at the same meteorological conditions in 10th of Ramadan City, Egypt (30.2927° N, 31.7423° E).

To ensure reliable comparative analysis, the experimental configurations were run simultaneously under equivalent environmental conditions. During the testing period, all the solar stills were filled with saline water to equal depth and irradiated by an identical intensity of solar radiation under identical ambient conditions. Before the experiments, systems were operated for several hours to reach thermal stability and provide stable baseline conditions. This approach would lessen the impact of external disturbances and allow for precise measurement to compare the performance improvements between the CSS, PCM-SS, and HSS.

### Experimental configurations

#### Case 1: conventional solar still (reference configuration)

The conventional solar still with a corrugated stainless-steel absorber plate in the baseline configuration has a projected surface area of 0.425 m². The basin liner is coated with black paint to optimize the efficacy of solar energy absorption. It is made of 1 mm galvanized iron. The use of blue foam in comprehensive thermal insulation prevents heat losses from the basin liner to the ground foundation and the atmospheric environment.

The transparent cover structure is performed by a 30 angular inclination of the 4 mm acrylic material. This material was selected for its excellent optical transmission, ease of use, and long-term performance. There is good silicon sealing on the base; it’s really very solid and durable, thereby preventing any vapor leakage.

The system geometry includes a front glass height of 10 cm, and the back glass is 70 cm from the absorber surface. There are three V-shaped channels, set at 45-degree angles to each other. The interior integrates a 4th collection channel linked with the internal wall. These passages allow the easy flow of collecting and carrying condensate to a storage means.

**Fig. 2:**
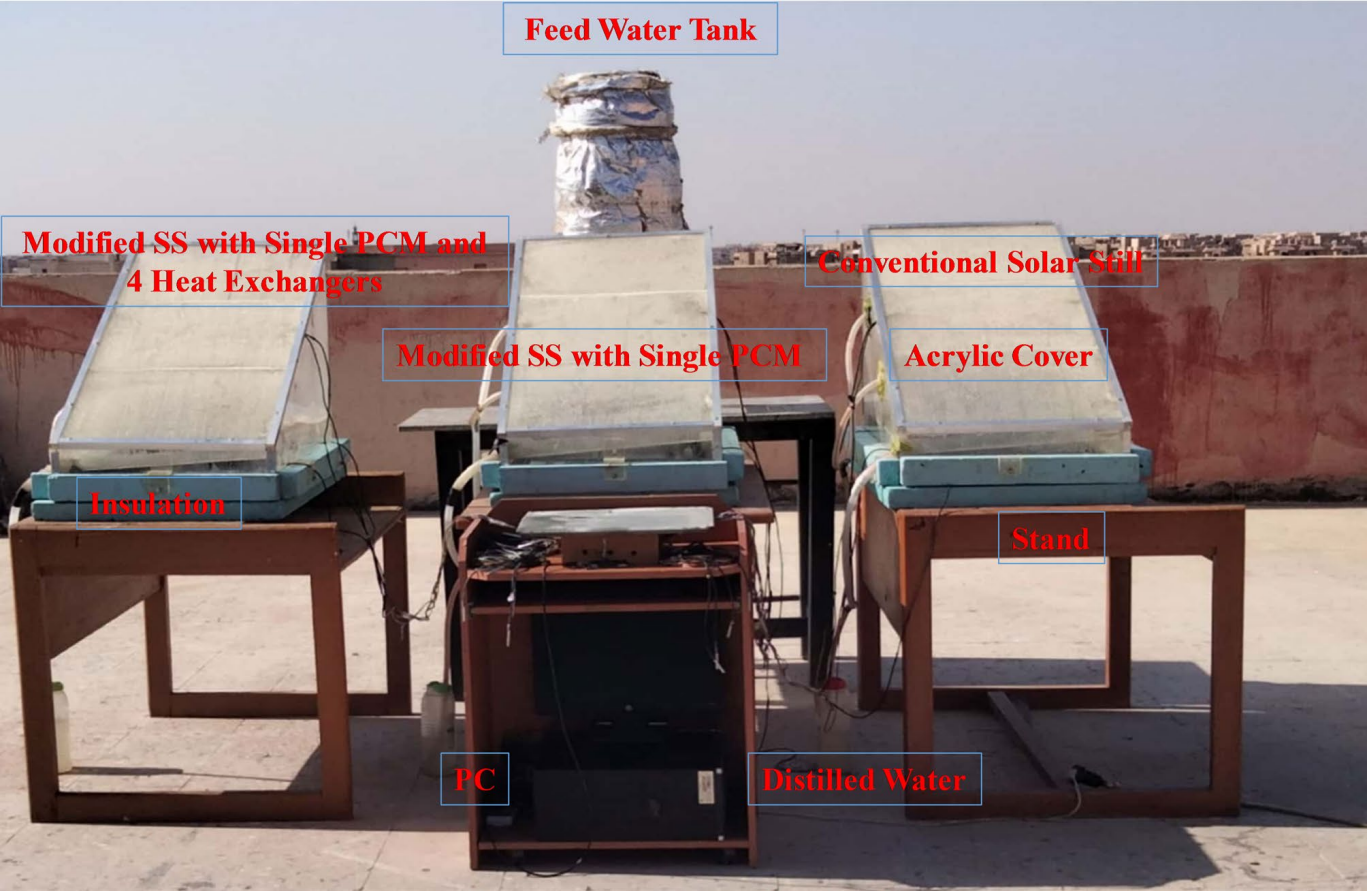
Photographic depiction of the experimental setup.

The plastic-piped water is conveyed to calibrated 1.5-liter storage bottles from each collection trough. This process facilitates distillate collection. Calibrated containers are employed to obtain an accurate measure and thus quantify freshwater production; this method is systematic. The simplified solar still design is shown in Fig. 2, showing the photograph of the complete experimental set-up.

#### Case 2: PCM-enhanced solar still

**Fig. 3:**
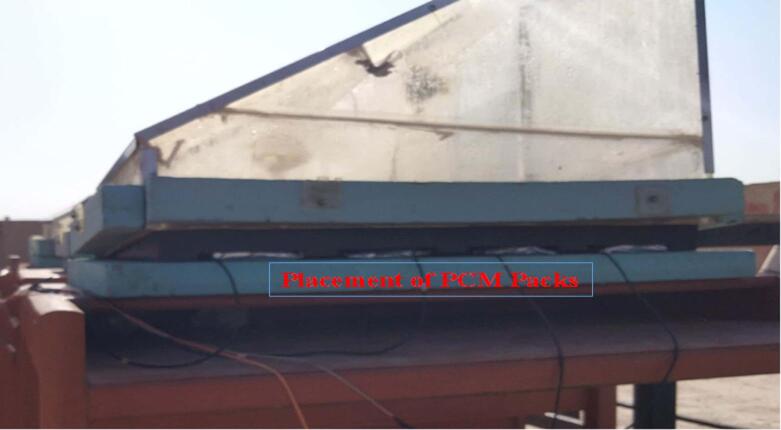
Placement of PCM packs in the configuration.

PCM storage containers are suitably placed underneath the absorber plate in the second configuration and used as a thermal energy reservoir. The PCM containers are fitted exactly into holes specifically cut out at the corrugated surfaces under the basin liner, forming a full thermal storage matrix as depicted in Fig. 3.

**Fig. 4:**
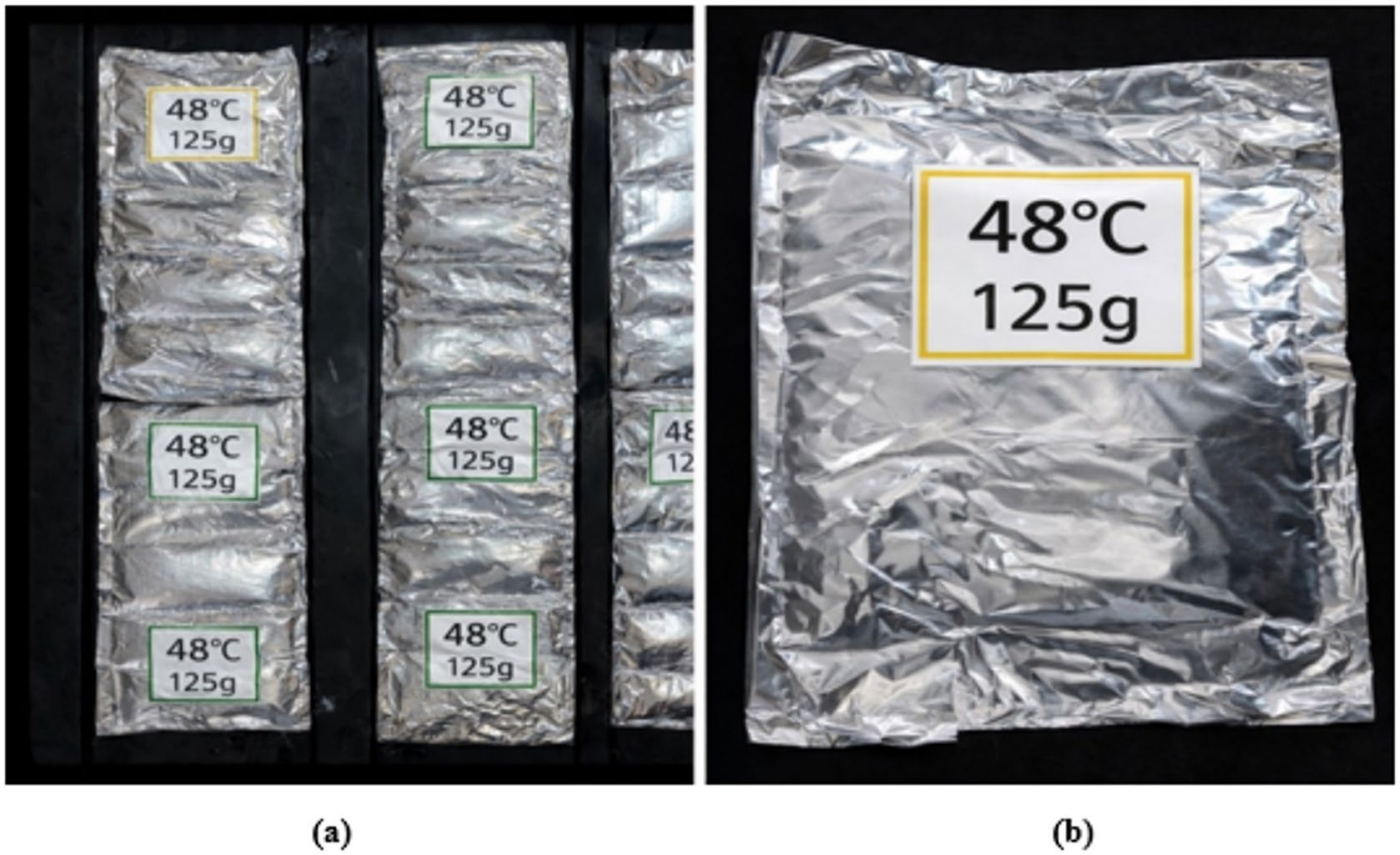
**(a)** PCM packs underneath the absorbent plate **(b)** The sizes of the PCM pack.

The Rubitherm© SP 48 was chosen due to its phase change temperature of 48 °C, as the PCM properties were based on ideal melting point characteristics. In addition, this temperature range provides a continuous heat release that will occur during low or no solar incident and efficient energy storage during high solar irradiance. Every single PCM container (12.5 × 15 cm²) holds 1.25 kg of PCM material. The optimal loading conditions are obtained through systematic variation of the total PCM mass in different test runs (0.5, 1, 1.5, 2, 2.5, 3, and 3.5 kg). Both the thermal conductivity and structural stability are enhanced with the help of aluminum coating. The specific dimensions of individual PCM pack holding structures are illustrated in Fig. 4, which also depicts the detailed positioning of PCM packs beneath the absorbent plate.

#### Case 3: PCM-enhanced solar still with heat exchange system

**Fig. 5:**
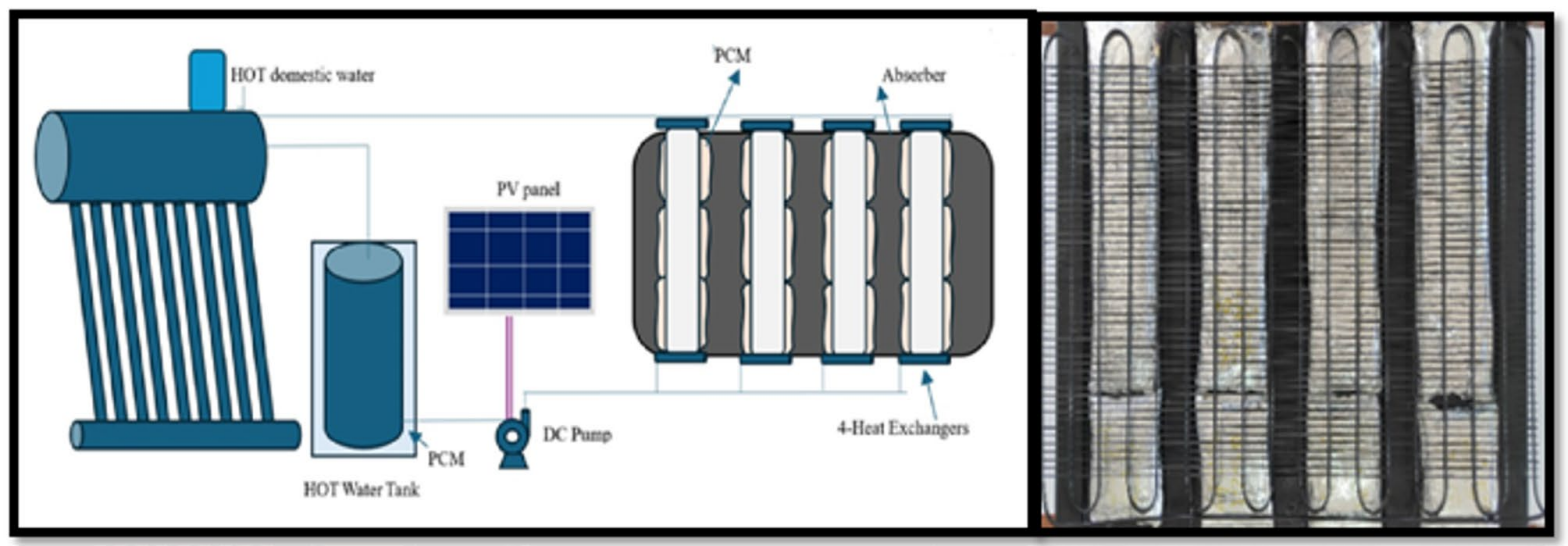
Schematic of solar stills with PCMs and heat exchangers.

The third configuration is the most sophisticated arrangement, as it includes an integrated heat exchange system and PCM thermal storage. Thermal energy exchange between the solar still system and an independent hot water storage tank is accomplished with four heat exchangers. The DC pump is powered by its own independent solar panels to provide circulation. Figure 5 Full schematic system and the picture of solar still with PCMs and heat exchangers^14,22^. With a coupling between PCM and active heat transfer incorporated in it, the hybrid system could enhance its energy utilization efficiency and thus may improve overall system performance and prolong the working period.

### Phase change material specifications

In the experimental study, commercially available PCM (Rubitherm© SP 48) was used due to its good thermophysical properties, which are suitable for this application. Overall mechanical and material specifications are given in Table 1:

**Table 1:** Further specifics concerning the PCM that underwent operation at 48 °C.

Specification	Rate
Melting temperature	48 °C
Density of liquid/solid	1200/1300 kg/m^3^
Specific heat in the liquid vs. solid	2000 J/kg °C
Latent heat of fusion	190,000 J/kg
Thermal conductivity of liquid/solid	0.5 to 0.7/0.5 to 0.7 W/m °C

The salt hydrate based PCM composition includes specialized additives to enhance thermal stability and prevent phase separation during repeated thermal cycling.

The melting temperature of the selected PCM (48 °C) lies within the typical operating temperature range of solar still basins under high solar radiation conditions. However, when the solar radiation values are low in winter months, the basin water temperature may not reach the PCM melting point. In these situations, the PCM would act partially as a sensible heat storage medium instead of a complete phase transition. During the discussion of seasonal applicability in the proposed system, this characteristic is considered.

### Operational principle and thermal dynamics

#### Daytime operation

**Fig. 6:**
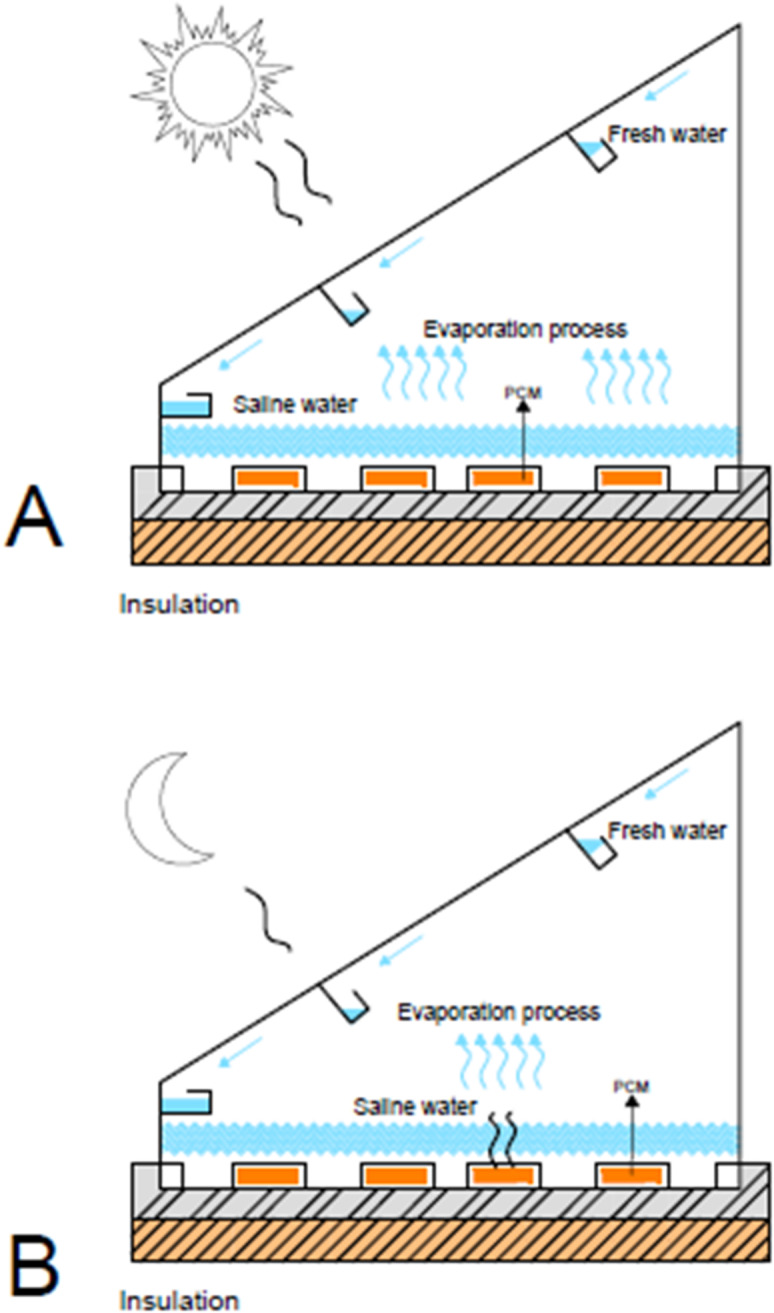
The PCM – advanced solar still experimental setup schematic (**A**) Day performance (**B**) Night performance.

In solar irradiance hours, the absorber plate absorbs thermal energy under a glass cover and provides it in two ways: particular heat transfer to saline water results in evaporation progress, and at the same time latent heat of PCM is stored. The collected distillate is transported via the inclined channel system (as shown in Fig. 6 (a), where, finally, evaporation of saline water to migrate at the colder glass cover condenses as distilled water.

#### Nighttime operation

Without solar availability, the PCM experiences controlled thermal discharge and discharges the stored energy to the absorber plate, then to the saline water. This continuous power feeding keeps the evaporation and desalination growing on at a low level to expand the working time of productivity after sunlight, as shown in Fig. 6 (b). Figure 6 also presents the day and night performance cycles from an operational point of view of the PCM-enhanced solar still in a graphic manner.

### Measurement and instrumentation

#### Operational parameters

The saline water depth remains consistent at 1 cm during the entire testing duration in all experimental runs. The feed seawater used in the experiments had a pH and total dissolved solids (TDS) measurements showing values ranging between 7.9 and 8.2 and TDS more than 35,000 mg/L, respectively, before desalination, whereas after distillation, the produced distillate had a pH reading ranging between 7.3 and 7.4 as per the WHO drinking water standards of potable water.

#### Environmental monitoring

A calibrated pyranometer tracks incident solar irradiance on a horizontal plane all the time, to supply valuable data for energy evaluation calculus.

#### Temperature measurement system

Waterproof temperature sensors are used to measure important system temperatures such as the acrylic cover, the basin liner, the saline water, the water vapor, the PCM, and ambient conditions.

The procedure used in determining the PCM temperature uses the six waterproof temperature sensors at different levels in the PCM storage container. The final values are calculated as the average of the readings captured. A centrally positioned sensor is used to monitor the temperature of the basin liner, while a dedicated submerged sensor is used to monitor the temperature of saline water. Two more sensor sites are used to monitor the temperatures of the inside acrylic.

All temperature sensors are connected to a data recording system (PSB, suitable for high temperatures) for reliable measurements during the experiments. Figure 1 Schematic architecture of the waterproof temperature sensor arrangement, whose purpose is to measure the temperature everywhere in the system.

#### Production measurement

Production of the fresh water is in liters monitored hourly by calibrated containers with a volume of 1.5 L. All measurements taken are stored automatically by the incorporated data recorder system (hourly).

### Experimental protocol and data collection

A full-day cycle is adopted by the complete data-collecting method from 08:00 AM to 10:00 PM for obtaining a full-charging phase and discharging stage of energy, which is mandatory to realize the thermal natural activities of PCM. To minimize differences in sun intensity and allow similar comparison among experimental trials, all experiments were conducted daily for five subsequent days (August 22–26, 2025) with constant weather conditions.

#### Pre-experimental preparation

As follows are the procedures for conducting experimental work:

1. It is of utmost importance that all preparations are made before experimental measurements are taken. This involves putting water into the basin a few hours before the readings are taken. The basin must be filled with water and monitored for the proper amount and depth.
2. The PCM volume, measuring instruments, and other applicable factors will be assessed in accordance with the nature of the case under investigation. To guarantee that the PCB registers the readings, it is crucial to record the measurements simultaneously during each measurement. During each measurement, the pyranometer will register the solar energy.
3. Maintain a record of the quantity of pure water that has been collected.
4. For each stage, repeat steps 3 and 4.
5. The measurements for the following day are conducted by repeating Step 1. After the completion of all daily measurements, the solar still should be prepared for the first reading day by cleaning the acrylic covering and ensuring that all insulated components are correctly insulated to prevent leakage, etc.

Although the experiment was performed over a relatively short five-day period during August, the selected test days provide relatively stable summer meteorological conditions with low variability in solar radiation and ambient temperature. As a result, the data obtained serves as an accurate reflection of the system’s performance at peak solar availability. The economic and environmental assessments reported later in this study use extrapolation based on standard economic evaluation methods that were widely used in the solar desalination research community. However, these results ought to be understood as scenario-based estimates instead of actual long-term metrics.

### Uncertainty analysis and error assessment

The maximum possible uncertainties in the calculated power output and other performance parameters were evaluated using the uncertainty propagation method proposed by Robert J. Moffat^33^. This method allows us to systematically determine the errors in the experiment because of measurement inaccuracies.

The uncertainty calculation was done by considering the accuracy specifications of the measurement instruments as per their manufacturer’s data, along with the minimum values of measurement output obtained during the experiment. Table 2 presents a summary of the accuracy specifications of the measuring instruments used in the experiments.

**Table 2:** Accuracy specifications of the measuring instruments used in the experiments.

Instrument	Range	Resolution	Accuracy
Waterproof temperature sensor	−10 to + 85 ◦C	9 to 12 bits	± 0.5 ◦C
Lutron TM-946 data logger Or PCB	−199.9 to 1370 ◦C	0.1 ◦C	± (0.5% + 1 ◦C)
A Pyranometer	0.1–1999.9 W/m^2^	0.1 W/m^2^	± 10 W/m^2^
Measuring beaker (calibrated flask)	0–1500 ml	5 × 10^− 5^ ml	± 0.01 ml

The analysis of uncertainty needs to carefully identify the different sources of error related to the measurement of the experiment’s variables.

If a calculated parameter S is a function of several independent variables $\:{x}_{1}$, $\:{x}_{2}$, $\:{x}_{3}$, …, $\:{x}_{\mathrm{n}}$​, the relative uncertainty in S can be estimated using the root-sum-square (RSS) method as follows:

1 $$\:\frac{\varDelta\:S}{S}=\sqrt{[{\left(\frac{\varDelta\:{x}_{1}}{{x}_{1}}\right)}^{2}+{\left(\frac{\varDelta\:{x}_{2}}{{x}_{2}}\right)}^{2}+\dots\:+{\left(\frac{\varDelta\:{x}_{n}}{{x}_{n}}\right)}^{2}}]$$

where $\:\varDelta\:{x}_{1}$, $\:\varDelta\:{x}_{2}$, … represent the absolute uncertainties related to the different variables of the experiment. On the other hand, the corresponding percentage uncertainty of the different variables can be represented as ($\:\frac{\varDelta\:{x}_{1}}{{x}_{1}}$), ($\:\frac{\varDelta\:{\mathrm{x}}_{2}}{{x}_{2}}$), etc. By considering the specifications of the different instruments used to collect the experiment’s data, the percentage uncertainty related to the measurement of the temperature (T), the volume of the distilled water produced (V), and the solar irradiance (I) were computed as ± 0.5%, ± 0.01%, and ± 0.1%, respectively. By applying the uncertainty propagation method to the governing equations related to the performance of the system, the percentage uncertainty related to the computed energy efficiency/exergy efficiency was computed as ± 0.5%.

The low level of the percentage uncertainty computed for the experiment’s results indicates that the different performance indicators computed from the experiment’s results are sufficiently reliable. This demonstrates that the different errors related to the measurement.

### Thermodynamic and economic evaluation methodology

The proposed solar still systems were evaluated by the 4E analysis framework, namely energy, exergy, economic, and environmental analyses. The energetic efficiency is defined as the ratio of the useful latent heat related to freshwater production to the total incident solar radiation on the basin area. Exergy efficiencies were determined considering the useful evaporated water exergy over the incoming solar radiation exergy.

The subsections below show and discuss governing equations for the energy, exergy, economic, and environmental analyses.

## Results and discussions

The error bars shown in the main figures represent the uncertainty of the respective measured or calculated parameters. The estimated uncertainties were based on the accuracy of the instrument and also propagation uncertainty analysis as described in Sect. 2.7.

### Environmental conditions and validation of tests

**Fig. 7:**
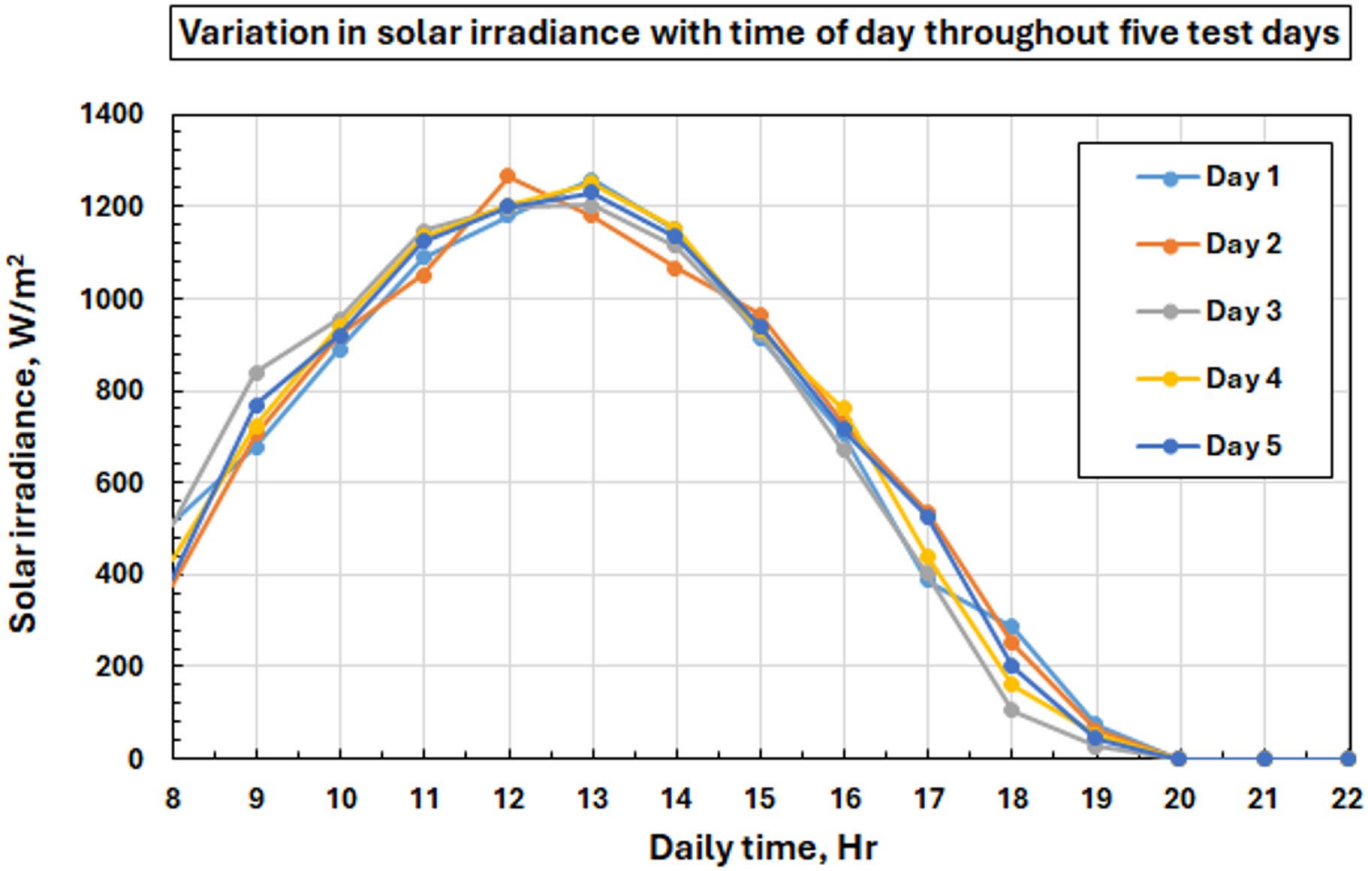
The solar radiation on five test days relative to the daylight time at the hour of the day.

Figure 7 shows the solar irradiation curves of five test days that occurred in August 2025 in order to emphasize the homogeneity of environmental conditions, fundamental to achieving reliable comparison analysis. The response of solar irradiance. The analyses, Fig. 3, indicate that solar radiation shows a normal bell-shaped curve with the maximum value of 1257 W/m^2^ at the time of solar noon. The average daily solar irradiance measurements were quite constant during the test period: 608.5, 607.4, 606.7, 611.8, and 613.1 W/m² with a small variation of ± 0.5%.

**Fig. 8:**
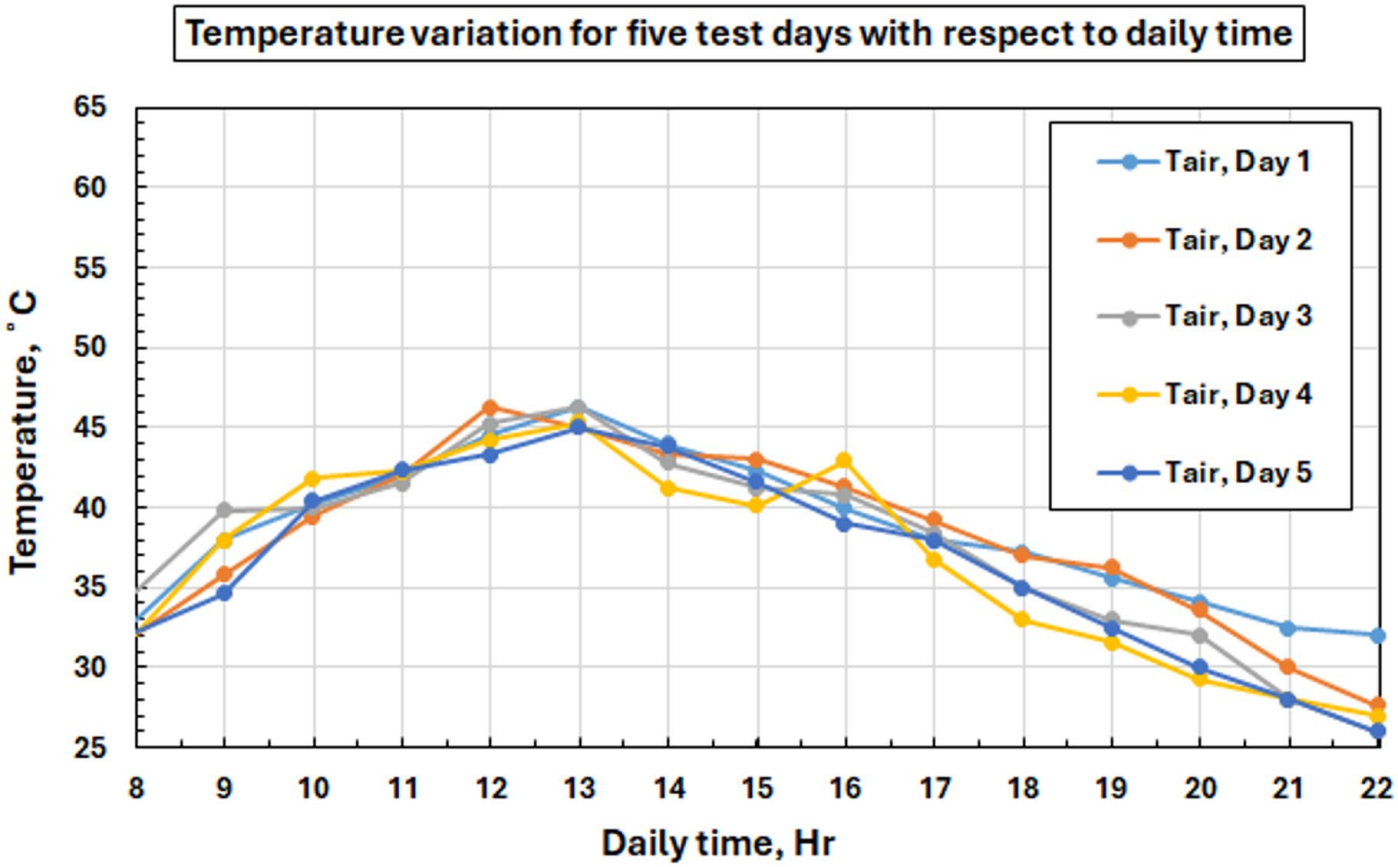
Variation of ambient temperature with time of day throughout five test days.

The daily variation of in-building temperature is illustrated in Fig. 8, indicating that the thermal environment was relatively stable during the measuring day, and therefore, our data analysis is reliable for performance evaluation. The temperature patterns are very smooth and regular: there is a rise in the morning to about 46 °C at 13:00, after which they fall again. Environmental temperatures were an average of 36.77–38.64 °C daily, which are nominal levels for summer temperatures in the area earmarked for 10th Ramadan City. The experiment remains stable when similar heating distributions occur on all testing days.

### Analysis of thermal performance

#### Temperature characteristics of conventional solar stills

**Fig. 9:**
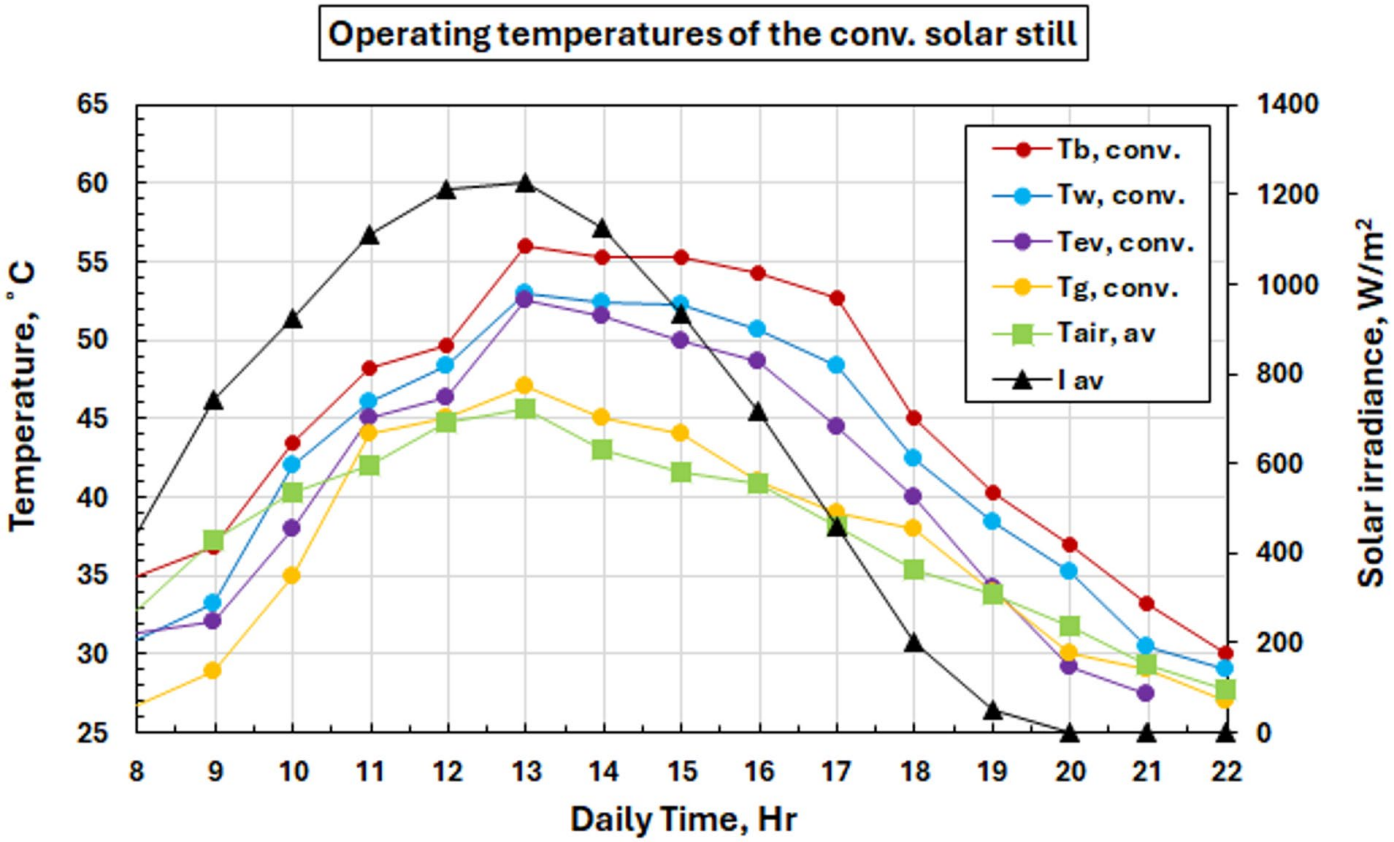
Solar radiation and temperature variations for the solar still with conventional solar still.

The basic thermal performance of a solar still system without any modifications is presented in Fig. 9. The graph emphasizes the clear coordinate process of solar radiation intensity and temperature in systems with high values at noon. The conventional method reached maximum temperatures of 54.3 °C for basin water, 50.7 °C for saline water, and 52.2 °C for vapor, with glass temperatures ranging between 27 and 47 °C. The temperature gradients are consistent with expected thermal behavior: gradually increasing in the morning as solar radiation intensifies, peaking around midday, then dropping during the afternoon and evening hours.

The temperature differences from the various parts of the system indicate good internal prototype structure heat transfer performance. Basin waters are always hotter than saline water because they receive direct solar energy (through a dark substrate) while in the glass and are intermediate since heating by sunlight and cooling by air are balanced.

#### PCM-enhanced system thermal dynamics

**Fig. 10:**
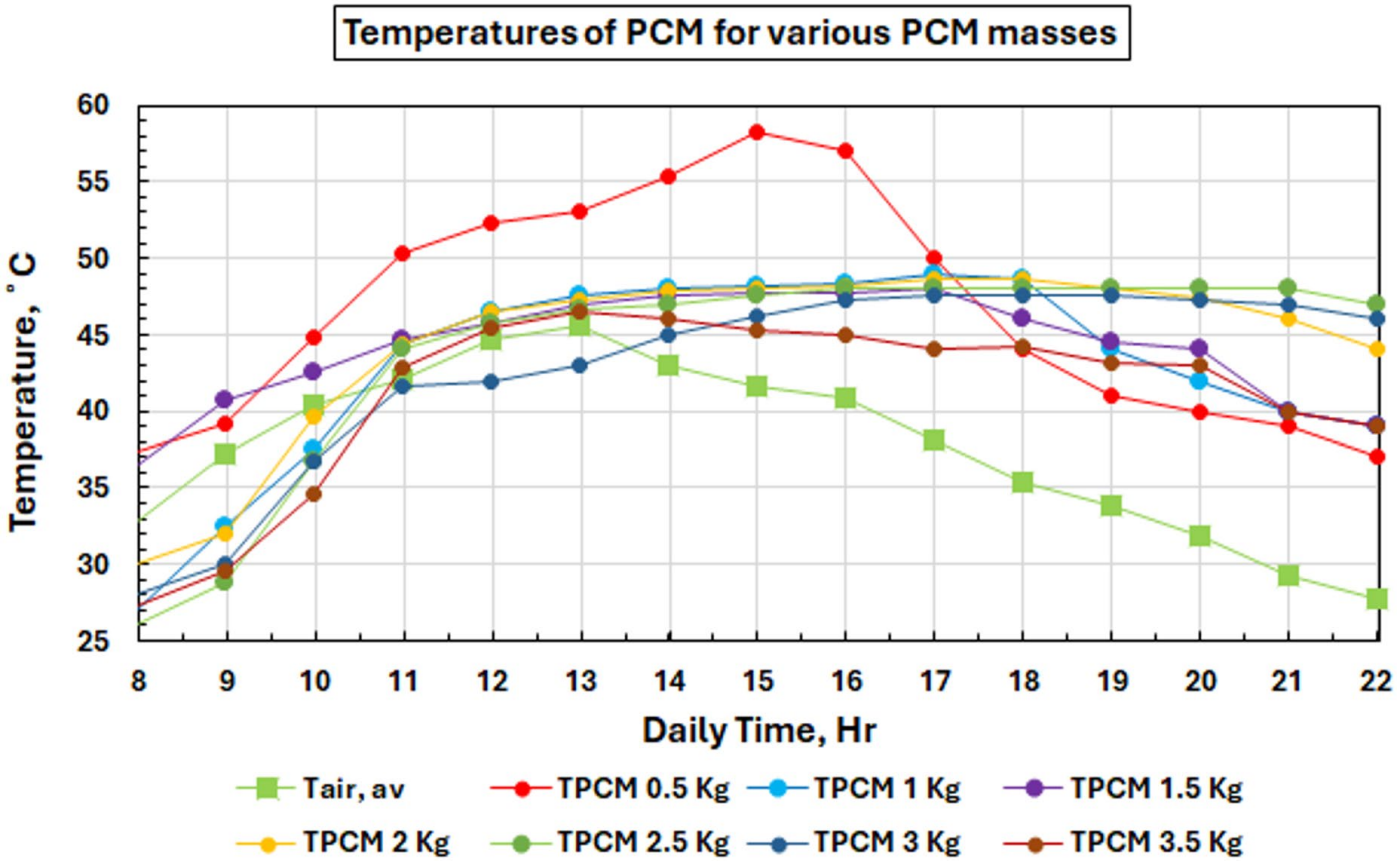
Ambient temperature variation and PCM temperature for different PCM masses.

Figure 10 illustrates a comparison between the temperature profiles of PCM loaded with different mass loads (0.5–3.5 kg) and those of environmental-temperature conditions. From 9:00 to 12:00, the initial temperature increases briefly due to solar radiation; from 12:00 to 20:00, the PCM remains solid. Thus, this stabilization time represents the total melting of the PCM and, hence, the transition from phase change energy absorption to sensible heat storage, which optimizes the efficiency of using 48 °C as a reference melting temperature for operational use.

**Fig. 11:**
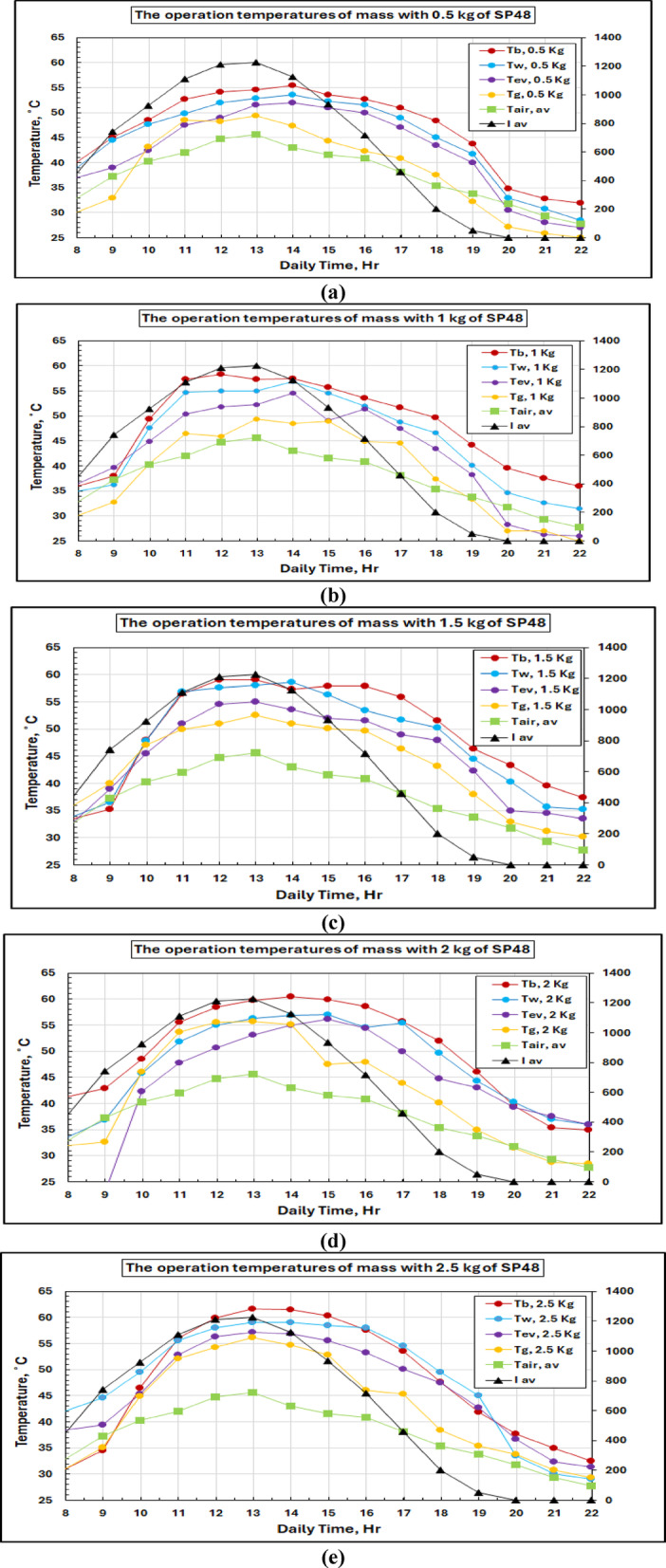

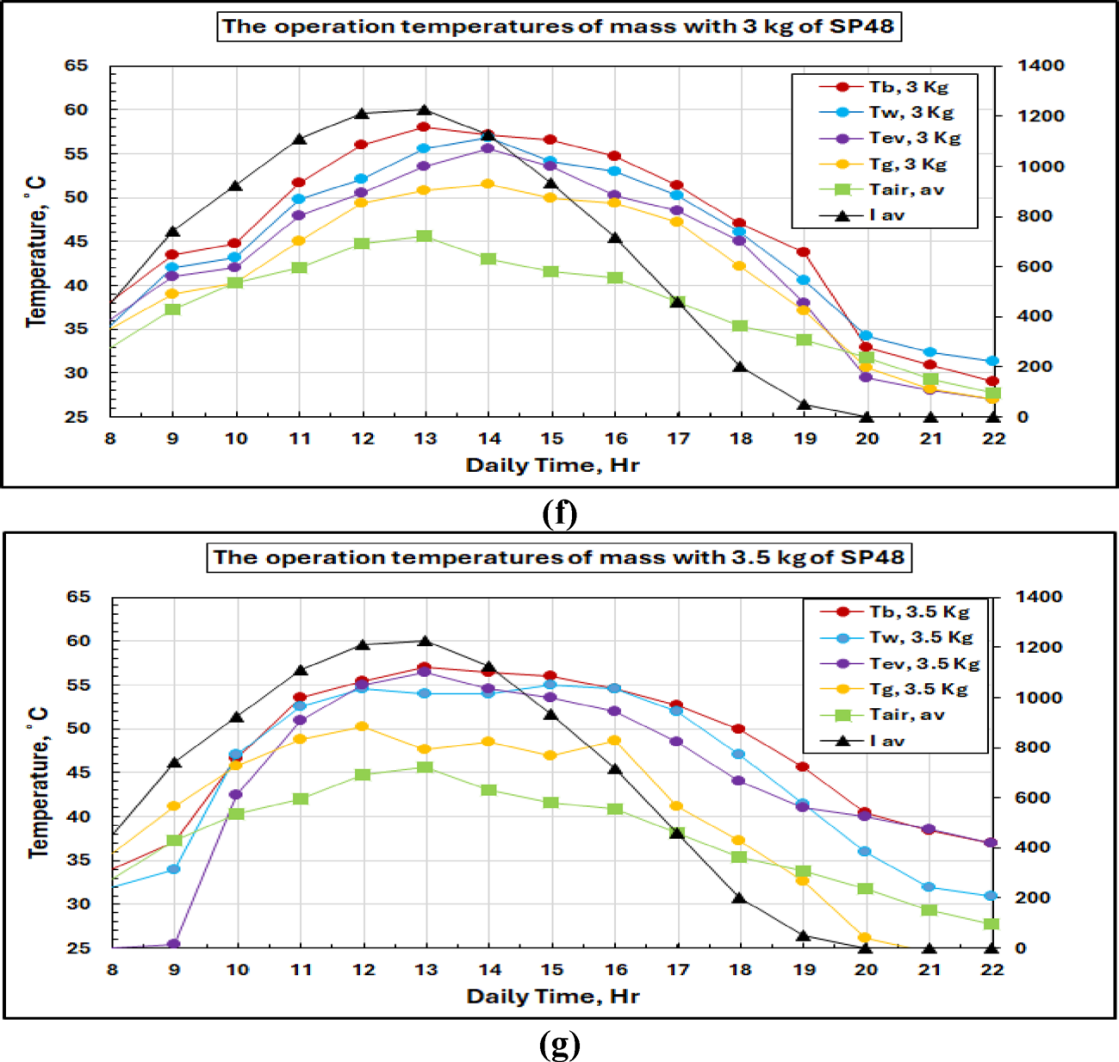
Solar radiation and temperature variations for the solar still with PCM at different masses of SP 48.

Thermal analysis for all PCM masses was performed, and the details are presented in Figs. 11(a-g); from this thermal study, it is also verified that the performance was very satisfactory at a 2.5 kg load condition. The peak system temperatures, found from the graphs, were 61.6 °C (basin water), 59 °C (saline water), 57.1 °C (water vapor), 56.2 °C (glass cover), and 45.6 °C with PCM of mass 2.5 kg for a maximum insolation value of 1200 W/m². Higher working temperatures of the order of 1808 C compared to conventional systems indicate that PCM thermal storage is an effective means for maintaining elevated working temperatures successfully. The temperature response of PCM has three stages as shown in the plots: initial sensible heating of solid PCM, isothermal phase change at 48 °C (melting point) and further sensible heating of liquid PCM. Such behavior confirms the correct choice of PCM and validates the thermal storage system.

#### Comparative thermal analysis

**Fig. 12:**
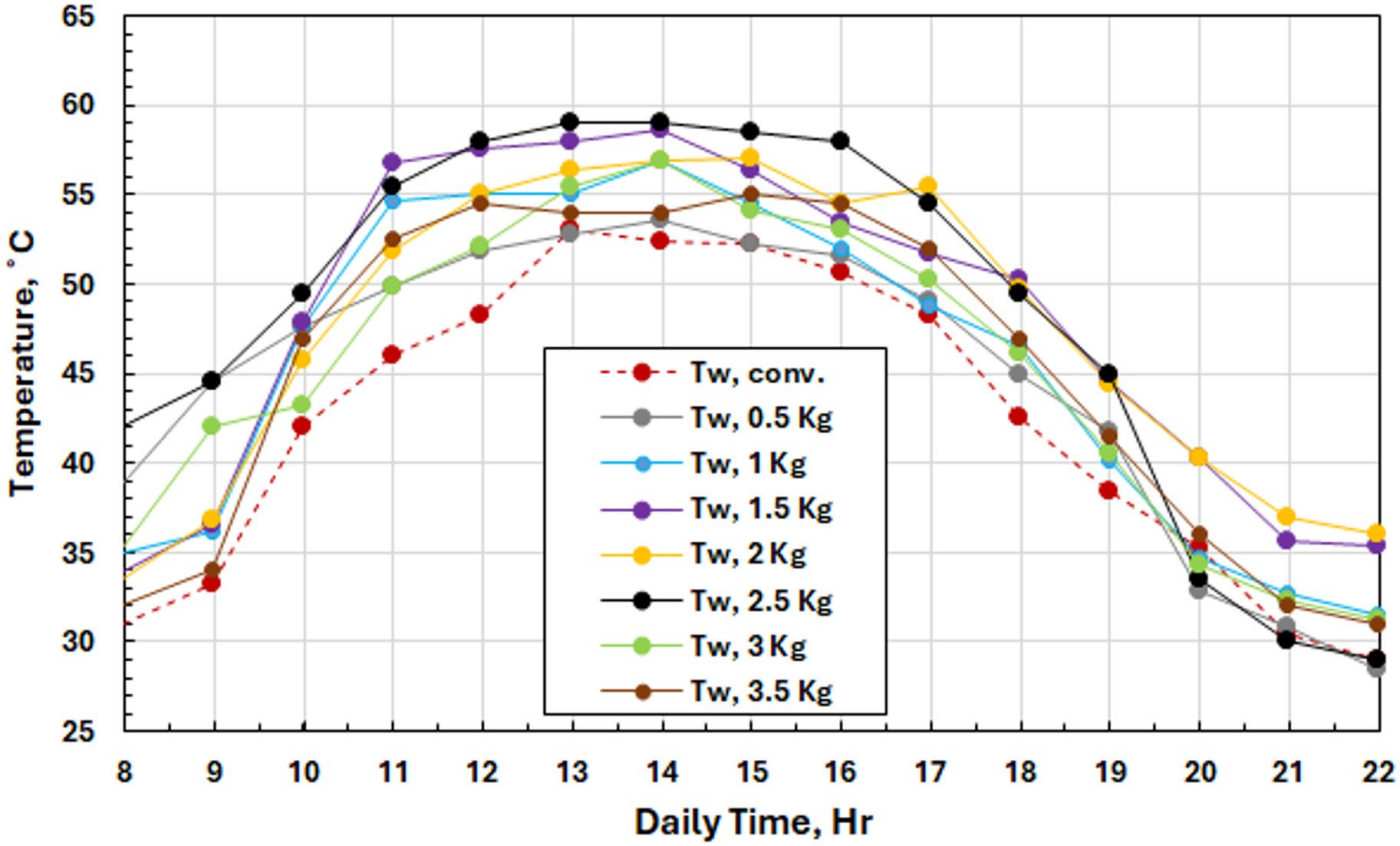
Water temperature variations for both the solar still with PCM at different masses and conventional.

Such thermal enhancement due to the addition of PCM can be highlighted in Fig. 12 when compared with all tested configurations. In early morning, which is when traditional systems are further populated with higher water temperatures by this time (where there is an effect from direct sun only and no storage) The basin water temperature of PCM augmented systems starts to rise significantly from 16:00 where the peak of a basin water temperature for the 2.5 kg system occurs at 13:00, which is reached as high as 59 °C. The thermal energy storage mechanism has to be highlighted during intervals of reduced solar irradiation, as the heat stored in PCM maintains high temperatures for saline water, contributing to a productive operating period greater than its ordinary operational limit. This greater retention of heat is reflected in the graph during twilight hours.

**Fig. 13:**
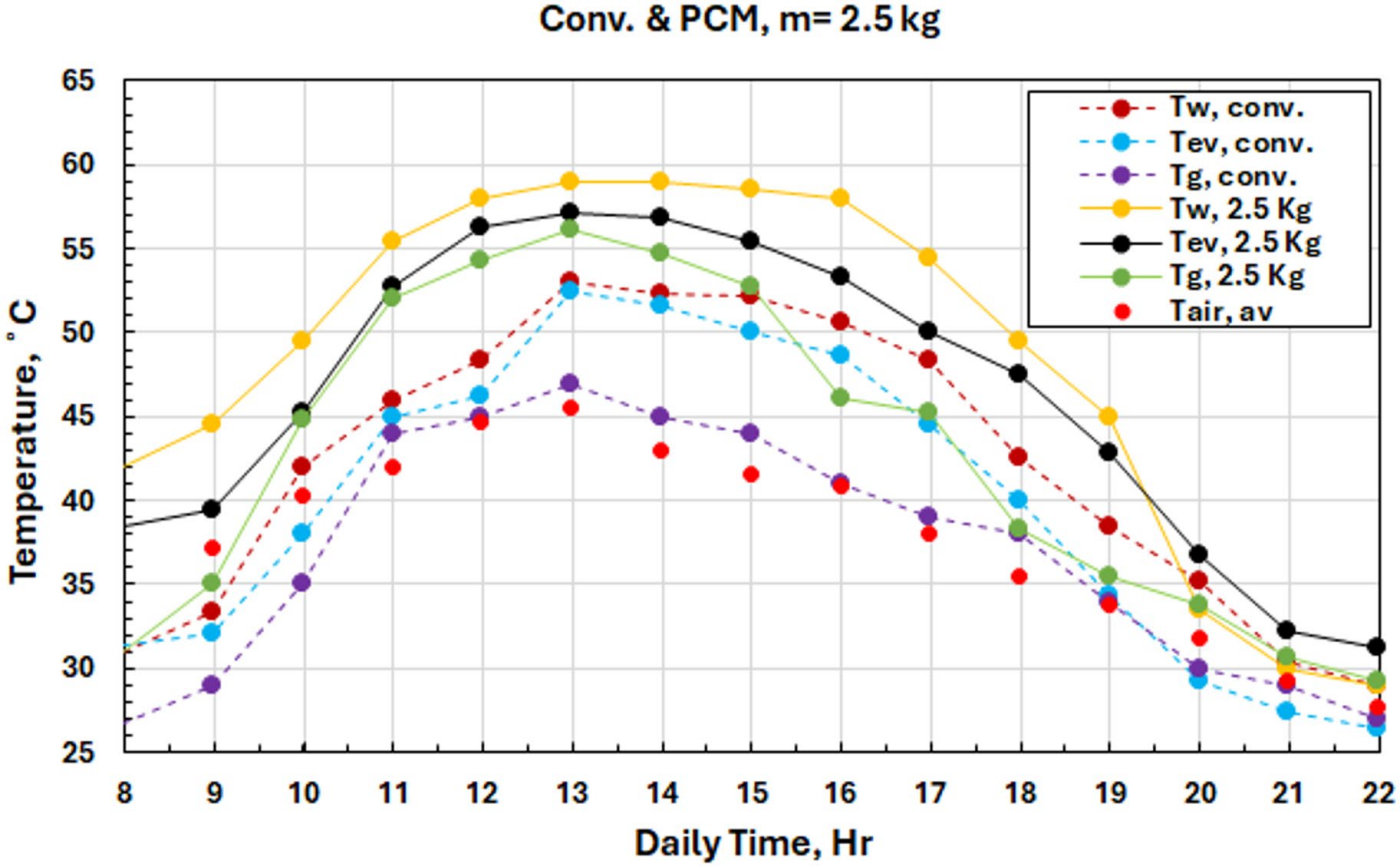
Temperature variations for both the solar still with PCM at mass of 2.5 kg and conventional.

Figure 13 presents comprehensive temperature comparisons between the ideal 2.5 kg phase change material system and the typical design. The results indicate that the PCM-enhanced system consistently sustained water temperatures 6–10 °C above those of conventional systems during the operational duration. The water vapor temperatures ranged from 31 to 57 °C, as compared to 26–52 °C in conventional systems, while glass cover temperatures had improved ranges of 29–56 °C compared to the ranges of 26–47 °C, indicating enhanced total thermal dynamics.

### Freshwater productivity

**Fig. 14:**
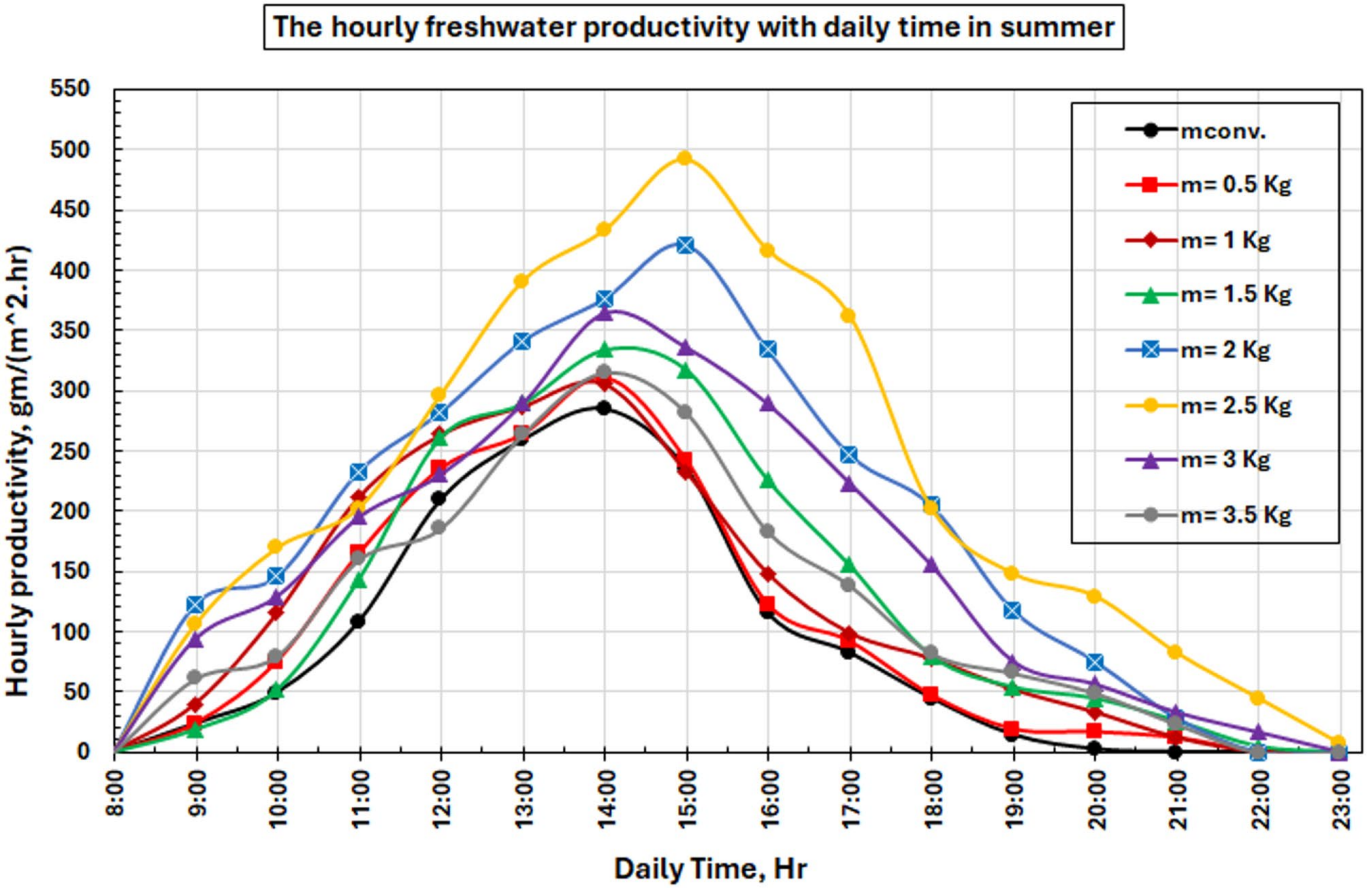
The rate of hourly freshwater productivity with time for the studied cases.

Figure 14 depicts the temporal fluctuations of hourly freshwater productivity across all evaluated configurations. The graph illustrates clear production patterns that corroborate the PCM enhancement process. Hourly productivity curves exhibit a progressive ascent from 8:00 to 13:00, with peak production roughly two hours post-maximum solar intensity, attributable to thermal lag effects between incident solar energy and the heating of brackish water. The performance data show that traditional systems first overtake PCM-enhanced ones in the morning when direct sun heating without thermal storage effects is prevailing. PCM systems are observed to maintain higher freshwater productivity than their solar contribution after 13:00, as the stored thermal energy still thermal energy to offset decreasing solar input, resulting in extended productive time and higher overall yields.

The noted improvement in the freshwater productivity of the PCM-augmented solar still can be ascribed to an increased thermal buffering action provided by the phase change material. The absorbed thermal energy is stored in PCM in the form of latent heat during peak solar radiation hours. This accumulated energy is slowly released when the sun’s intensity decreases or after sunset, ensuring a higher basin water temperature than that of traditional solar stills. Thus, the evaporation process lasts longer, and more cumulative freshwater is produced. Moreover, the thermal mass produced by incorporating PCM also smooths any significant temperature fluctuation in the basin environment, facilitating better heat and mass transfer in the system.

**Fig. 15:**
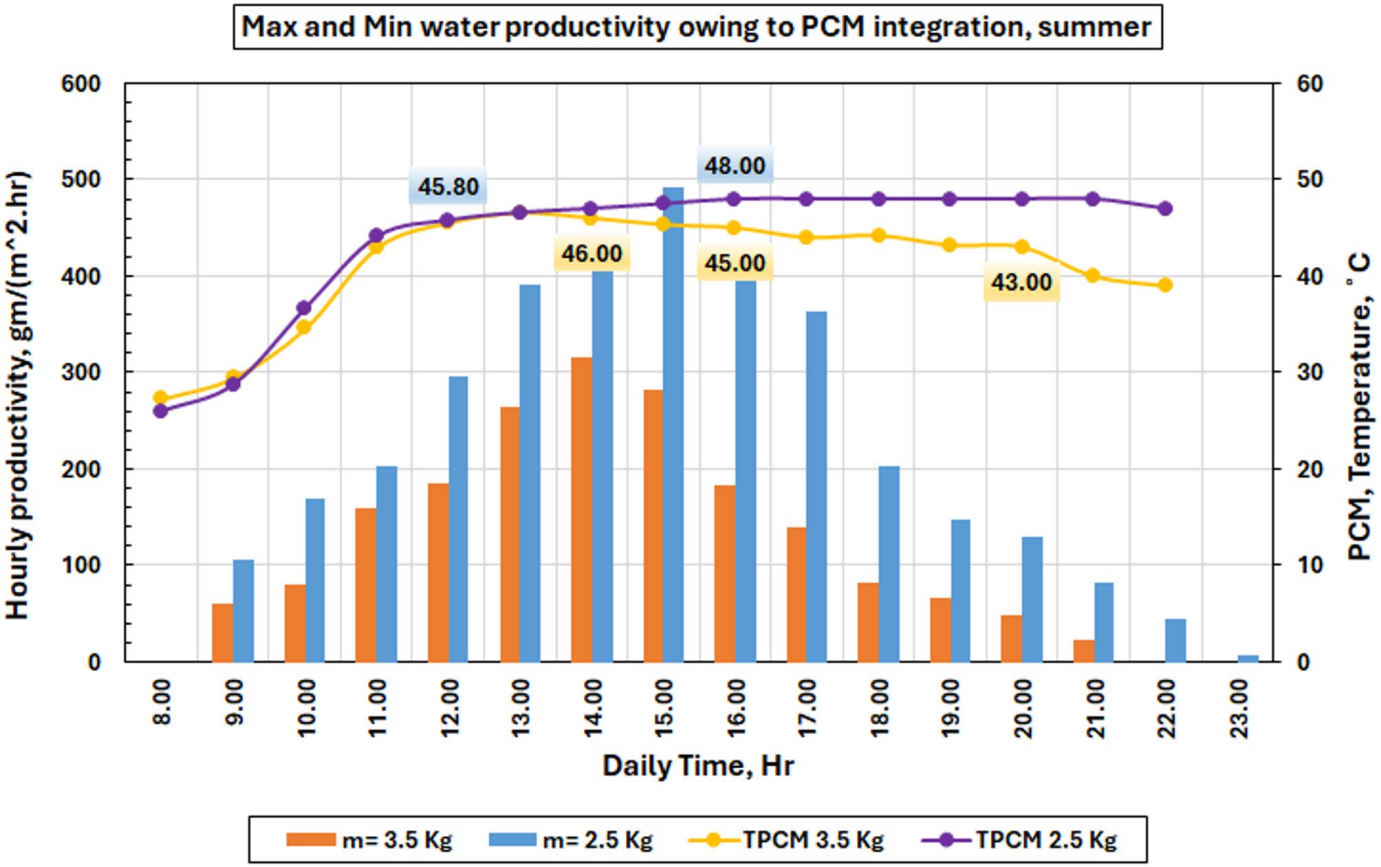
Time dependence of PCM temperature variations and the corresponding hourly rate of freshwater productivity for two different masses of PCM.

The evolution of the PCM temperature in function of the produced freshwater is presented for the two PCM masses that were most efficient (2.5 kg and 3.5 kg) in Fig. 15. The plot shows that the equipment from the 2.5 kg run is better as it has a higher average hourly production over the whole processing time. The optimum mass has its peak output at around 15:00, but the 3.5 kg system doesn’t do as well because the PCM does not melt completely, and thermal cycling is not perfect.

The performance drop at 3.5 kg is due to the ratio of solar energy available for charging versus the large PCM mass. As the PCM mass gets too high, absorbed solar energy is spread across a larger storage mass so that full melting does not occur during daytime. Thus, a portion of the PCM is in the sensible heating stage, and its latent heat storage capacity remains unutilized. This results in a lower effective amount of recoverable heat released during the late afternoon and evening, resulting in a small decrease in productivity increase as compared to the optimum case at 2.5 kg. The graph shows that the PCM temperature profiles are directly related to the amount of solar radiation coming in. They reach their highest point about noon and then slowly drop off till dusk. The 2.5-kilogram system had higher PCM temperatures because it was better at changing latent heat into sensible heat. This made the system more thermally stable and better at storing heat.

**Fig. 16:**
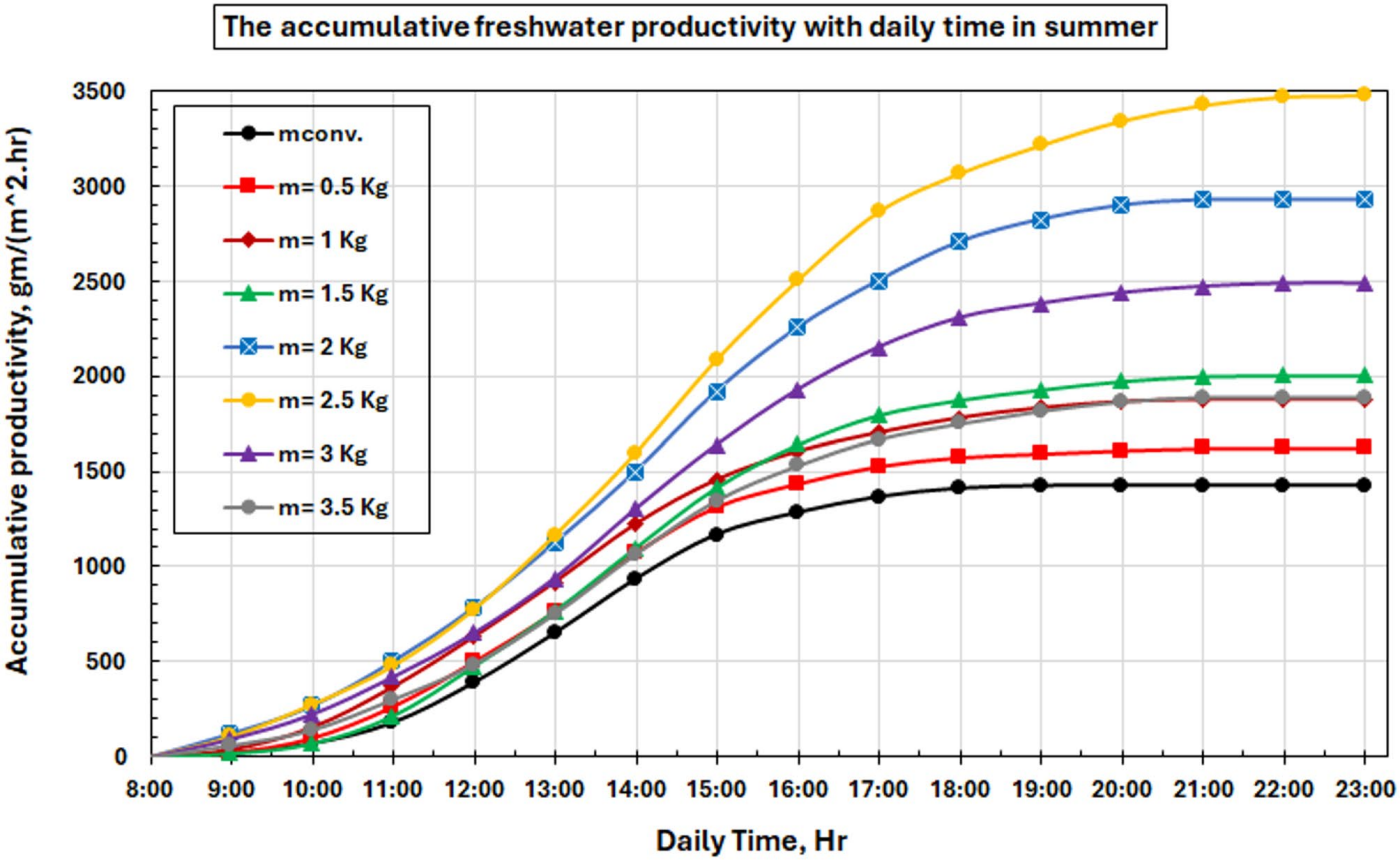
Accumulated freshwater productivity with time for the studied cases.

Figure 16 shows our most important experimental outcome: a comparison of cumulative freshwater productivity for the PCM-enhanced system with that of conventional systems. The graph tells us that the optimal 2.5 kg PCM-introduced system reached 2482 g m²/day as compared to those of conventional systems at 1428 g m²/day, which was a high enhancement of 73.8% per daily rate of production. Such a large improvement does indeed illustrate the potential of PCM as a heat storage medium for prolonging working hours. The productivity curves diverge significantly after 16:00 because, at this time, the heat released by the PCM compensates for the decrease in solar irradiation. Morning productivity losses in PCM systems are more than offset by later afternoon and evening output, resulting in a significant increase in the overall daily productivity.

**Fig. 17:**
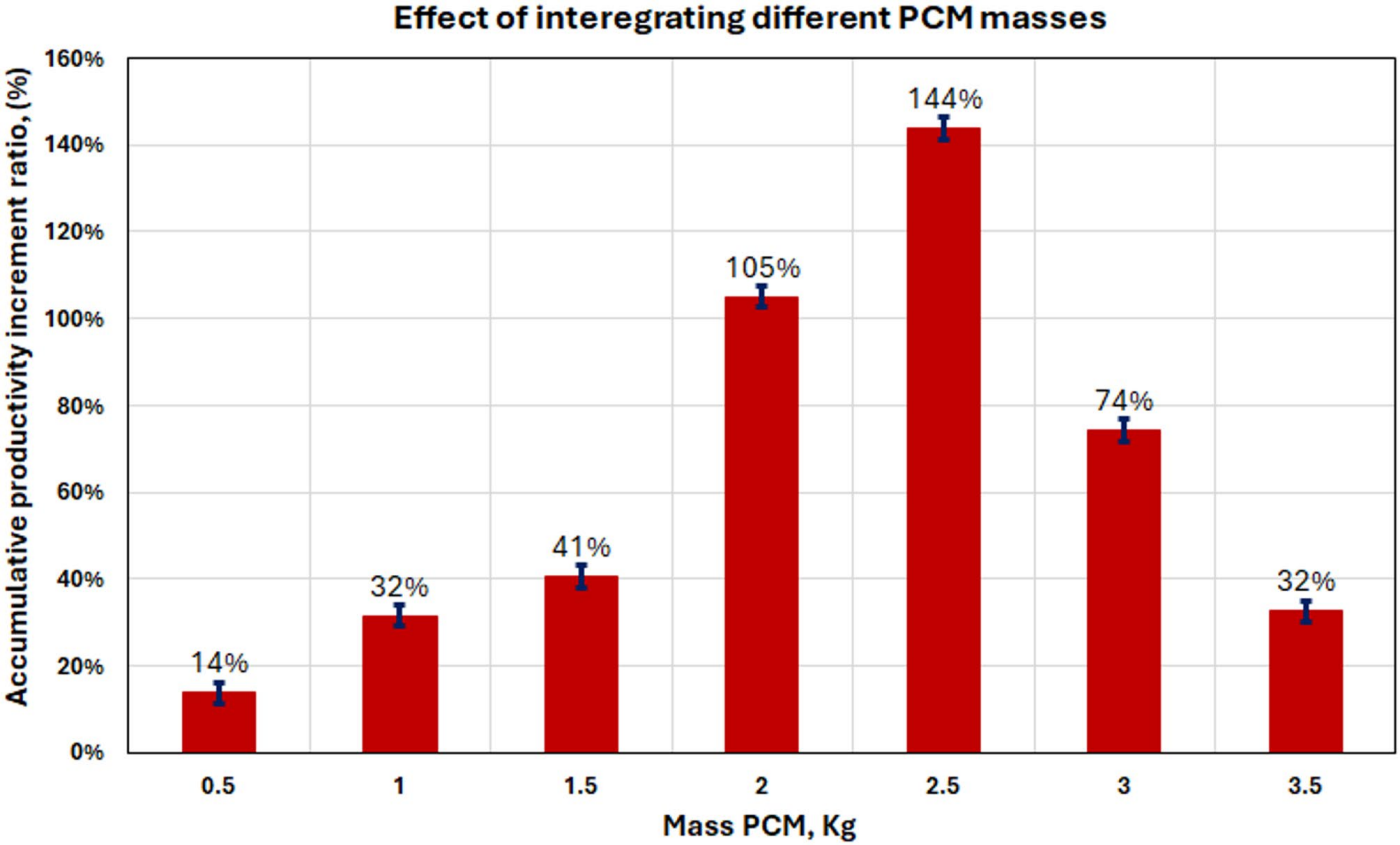
Accumulated freshwater productivity increment ratio (%) for various PCM mass.

Figure 17 shows the productivity increase for different PCM masses, which shows that there is a non-linear relationship between PCM loading and performance improvement. The bar chart indicates that work-rate enhancement is obtained at 14% (0.5 kg), 32% (1.0 kg), 41% (1.5 kg), 105% (2.0 kg), 144% (2.5 kg), 74% (3.0 kg) and, finally 32% (3.5 kg) That makes it clear that 2.5 kg is the optimal amount of weight to maximize performance gain.

### Theoretical evidence analysis

#### Energy analysis

For all investigated cases, the thermal efficiency of the solar still was evaluated to support further performance analysis and interpretation. This relation is utilized to obtain the solar still’s total energy efficiency^14^.

2 $$\:{\upxi\:}=\frac{{\mathrm{m}}_{\mathrm{d}}\:\times\:{\:\:\mathrm{h}}_{\mathrm{f}\mathrm{g}}}{\mathrm{A}\:\:\times\:\:\:\mathrm{I}\left(\mathrm{t}\right)}$$

Where I(t) is the total daily solar irradiation energy on the solar still in W/m^2^, A is the projected area of the solar still in m^2^, $\:{\:\mathrm{h}}_{\mathrm{f}\mathrm{g}}$is the latent heat of evaporation of water in J/kg, computed based on Eq. (3), and $\:{\mathrm{m}}_{\mathrm{d}}$ is the total daily freshwater productivity in kg/m^2^.

3 $$\:{\:\mathrm{h}}_{\mathrm{f}\mathrm{g}}=1000\:(2501.9-2.40706\:Tw\:+(1.192217\times\:{10}^{-3}\:{Tw}^{2})-(1.5863\times\:{10}^{-5}\:{Tw}^{3}\left)\right)\:$$

**Fig. 18:**
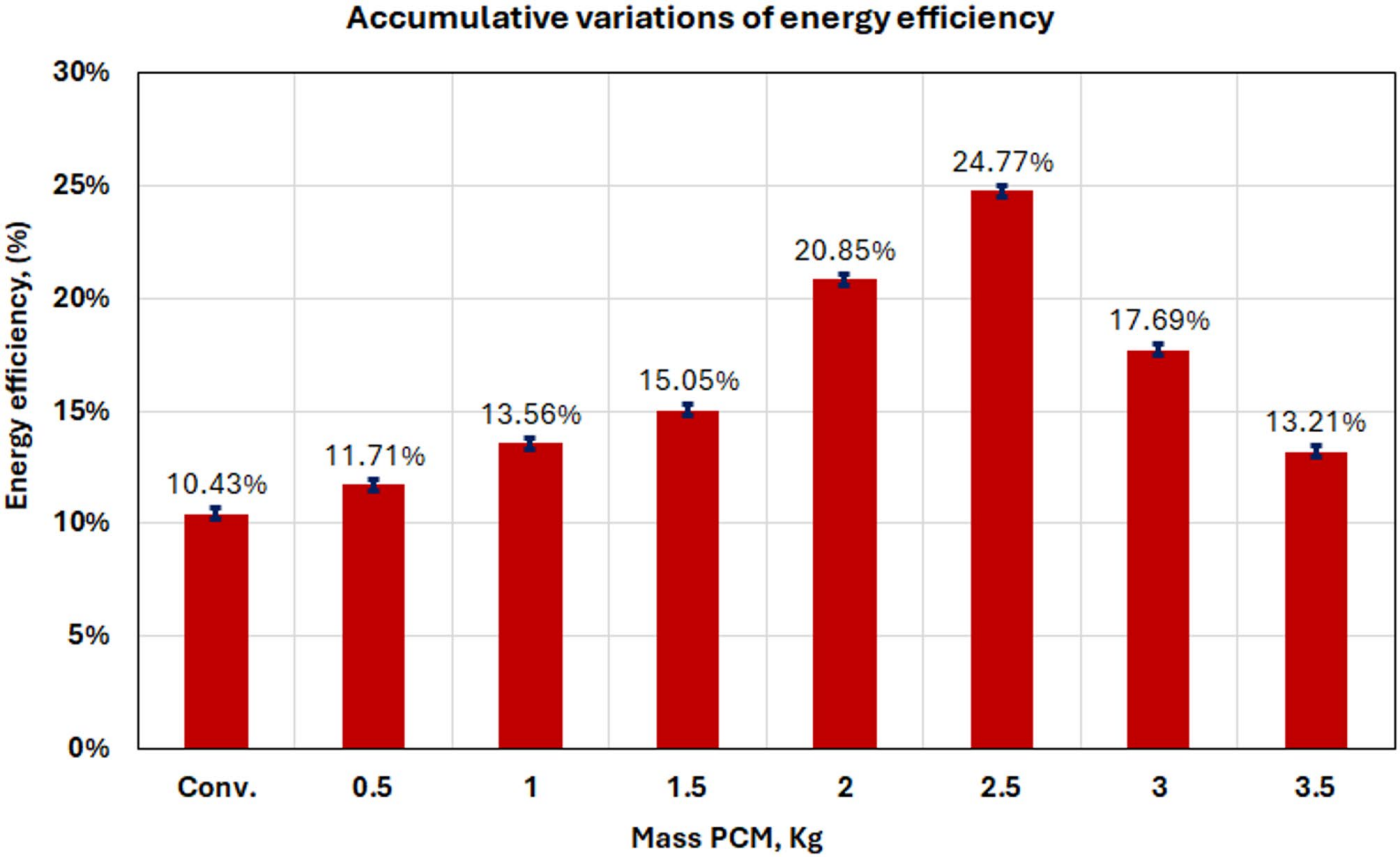
Average energy efficiency per day, for the cases considered.

The daily average energy efficiency assessment for all examined configurations is shown in Fig. 18. According to the bar chart below, it was found that the 2.5 kg PCM system reached peak energy efficiency (24.77%), which is significantly greater than conventional systems. The above efficiency is mainly due to better heat retention and lower thermal losses accomplished by reasonable-rate evaporation materials that PCM could store.

The enhancements in efficiency are directly associated with heightened heat gains and expanded temperature differentials between the glass cover and basin water, facilitating vigorous evaporation processes. Larger PCM masses showed decreased efficiency as their melting is incomplete or too much thermal mass exists relative to solar energy input.

#### Exergy analysis

By way of the second law of thermodynamics, we know that the exergy analysis function represents how useful work can be done with energy. The maximum amount of work which can be produced from a system as it comes into equilibrium with a given environment is called Exergy. The exergy balance, in general form, is written as^8^.

4 $$\:\sum\:{\mathrm{E}}_{\mathrm{x},\mathrm{i}\mathrm{n}}^{\circ\:}-\sum\:{\mathrm{E}}_{\mathrm{x},\mathrm{o}\mathrm{u}\mathrm{t}}^{\circ\:}=\sum\:{\mathrm{E}}_{\mathrm{x},\mathrm{d}\mathrm{e}\mathrm{s}\mathrm{t}}^{\circ\:}$$

The solar irradiance exergy is the exergy input to the solar still and is calculated by^20^,

5 $$\:{\Sigma\:}{{\mathrm{E}}^{^\circ\:}}_{\mathrm{x},\mathrm{i}\mathrm{n}}={{\mathrm{E}}^{^\circ\:}}_{\mathrm{x},\mathrm{s}\mathrm{u}\mathrm{n}}={\mathrm{A}}_{\mathrm{b}}\times\:\:\mathrm{I}\left(\mathrm{t}\right)\:\times\:\:[1-\frac{4}{3}\left(\frac{{\mathrm{T}}_{\mathrm{a}\mathrm{i}\mathrm{r}}+273}{{\mathrm{T}}_{\mathrm{s}}}\right)+\frac{1}{3}(\:\frac{{\mathrm{T}}_{\mathrm{a}\mathrm{i}\mathrm{r}}+273}{{\mathrm{T}}_{\mathrm{s}}}{\:)}^{4}]$$

Where $\:{\mathrm{T}}_{\mathrm{s}}$ is the sun temperature, 6000 K, $\:{{\mathrm{E}}^{^\circ\:}}_{\mathrm{x},\mathrm{s}\mathrm{u}\mathrm{n}}$ is the exergy input to the solar still from, and A_b_ is the effective area of the still basin in m^2^. $\:\mathrm{I}\left(\mathrm{t}\right)$is also the accumulated solar irradiance incident on the solar still in W/m^2^.

For a specific solar still, the product’s exergy production (distillate water) may be calculated using,

6 $$\:{{E}^{^\circ\:}}_{x,out}={{E}^{^\circ\:}}_{x,evap}=\frac{{{m}^{^\circ\:}}_{ev\:}\times\:\:{h}_{fg}}{3600}\left[1-\left(\frac{{T}_{air}+273}{{T}_{w}+273}\right)\right]$$

7 $$\:{\mathrm{h}}_{\mathrm{f}\mathrm{g}}=3.1615\left({10}^{6}-\left(761.6\:{\mathrm{T}}_{\mathrm{i}}\right)\right)\:\:\:\:\:\:\:\:\:\:\:\:\:\:{\mathrm{T}}_{\mathrm{i}}>70$$

$$\:{\mathrm{h}}_{\mathrm{f}\mathrm{g}}=2.4935({10}^{6}-947.79\:{\mathrm{T}}_{\mathrm{i}}+0.13132\:{{\mathrm{T}}_{\mathrm{i}}}^{2}-0.0047974\:{{\mathrm{T}}_{\mathrm{i}}}^{3})\:\:\:\:\:$$

8 $$\:\left\{\:\:\:\:\:\:\mathrm{W}\mathrm{h}\mathrm{e}\mathrm{r}\mathrm{e}\:{\mathrm{T}}_{\mathrm{i}}=\frac{{\mathrm{T}}_{\mathrm{w}}+{\mathrm{T}}_{\mathrm{g}}}{2}\:<70\:\right\}$$

Also $\:{\mathrm{h}}_{\mathrm{f}\mathrm{g}}$ is the latent heat of vaporization and $\:{{\mathrm{E}}^{^\circ\:}}_{\mathrm{x},\mathrm{e}\mathrm{v}\mathrm{a}\mathrm{p}}$ is the output evaporative exergy.

Exergy efficiency term is an expression string: $\:{{\mathrm{E}}^{^\circ\:}}_{\mathrm{x},\mathrm{i}\mathrm{n}}$ = Input exergy.

9 $$\:{\upxi\:}=\frac{{{\mathrm{E}}^{^\circ\:}}_{\mathrm{x},\mathrm{e}\mathrm{v}\mathrm{a}\mathrm{p}}}{{{\mathrm{E}}^{^\circ\:}}_{\mathrm{x},\mathrm{i}\mathrm{n}}}$$

**Fig. 19:**
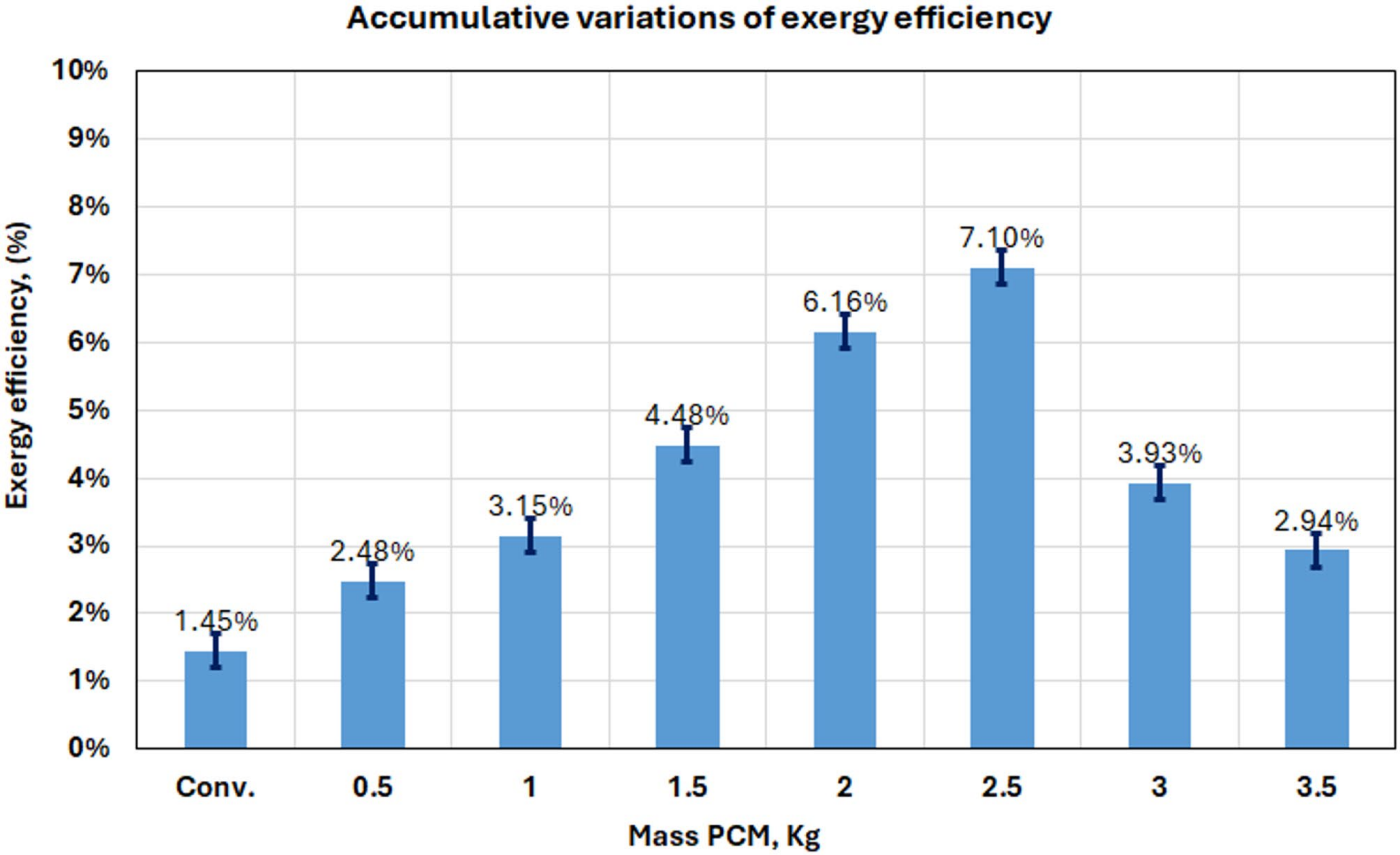
Daily mean exergy efficiency values for the cases considered.

Figure 19 illustrates an exergy efficiency analysis, offering a second-law thermodynamic evaluation of system performance. The graphic indicates that the 2.5 kg PCM design attained a peak daily exergy efficiency of 7.10%, due to the 16 °C temperature difference _b_etween ambient and basin water conditions. Such a big temperature gap allows for minimizing thermodynamic irreversibilities and maximizing system exergy efficiency. Improvements in exergy efficiency arise from various variables, including augmented condensed water volumes, improved latent heat usage, and optimal surface area exposure to solar radiation. The elevated salty water temperatures in PCM systems enhance exergy performance relative to traditional designs.

It is important to note that the thermal and productivity results presented in this study were measured during a five-day summer experimental campaign carried out under relatively stable meteorological conditions. Hence, the performance measured is reflective of how a system would work during peak solar availability in the summer. The economic and environmental indicators introduced in the next sections are extrapolated based on typical evaluation procedures widely adopted in the literature of solar desalination studies. In that sense, the following should be viewed as scenario-based estimates rather than direct long-term annual measures.

Calculations were then conducted to predict annual freshwater production according to the seasonal variation in solar radiation indicated above, which is a more realistic basis for economic and environmental assessments.

### Estimation of winter performance based on solar radiation data

The climatic conditions when the experimental study included August during peak summer, with high solar radiation intensity and ambient air temperature, where Solar still systems, which tend to achieve their maximum productivity under these conditions, owing to accelerated evaporation rates inside the basin. However, solar radiation and ambient temperature differ considerably during the year, which significantly impacts solar desalination systems in terms of thermal performance and freshwater production. As a result, assuming summer model parameters as representative of annual system performance can lead to an overestimation in productivity.

To solve this problem, a seasonal performance evaluation was carried out, depending on the changes in monthly average solar radiation. Previous studies have shown that more solar radiation correlates strongly with solar still productivity, so the ratio between months can reliably be used to estimate differences in freshwater production over a season^34^.

Table 3 shows representative monthly average solar radiation values considered when estimating seasonal changes in productivity. Such values are in accordance with typical solar radiation data described for climates similar to those analyzed in this work and often used in the performance analysis of solar desalination.

**Table 3:** Typical monthly average solar radiation and relative productivity ratio.

Month	Solar Radiation (kWh m⁻² day⁻¹)	Relative Radiation Ratio
January	3.1	0.46
February	4.0	0.59
March	5.1	0.75
April	6.2	0.91
May	6.7	0.99
June	6.8	1.00
July	6.7	0.99
August	6.8	1.00
September	6.0	0.88
October	5.0	0.74
November	3.9	0.57
December	3.2	0.47

Since solar still performance is mainly in proportion to the incoming solar radiation, expected freshwater productivity during months with less sunshine can be predicted using the following equation:

10 $$\:{P}_{winter}\text{}={P}_{summer}\times\:\:\frac{{I}_{winter}}{{I}_{summer}}$$

Where.

$\:{\boldsymbol{P}}_{\boldsymbol{w}\boldsymbol{i}\boldsymbol{n}\boldsymbol{t}\boldsymbol{e}\boldsymbol{r}}\text{}$​ = estimated winter productivity (kg.m⁻² day⁻¹).

$\:{\boldsymbol{P}}_{\boldsymbol{s}\boldsymbol{u}\boldsymbol{m}\boldsymbol{m}\boldsymbol{e}\boldsymbol{r}}$ = experimentally measured summer productivity.

$\:{\boldsymbol{I}}_{\boldsymbol{w}\boldsymbol{i}\boldsymbol{n}\boldsymbol{t}\boldsymbol{e}\boldsymbol{r}}$ = average solar radiation during winter months.

$\:{\boldsymbol{I}}_{\boldsymbol{s}\boldsymbol{u}\boldsymbol{m}\boldsymbol{m}\boldsymbol{e}\boldsymbol{r}}$​ = average solar radiation during summer months.

The average solar radiation is about 45–55% of the summer radiation level, as shown in Table 3. Thus, the freshwater productivity of the solar still during the winter session is anticipated to decline accordingly and may reasonably amount to nearly 45–55% of peak summer productivity. This estimation confirms the strong impact of seasonal climatic conditions on the performance of solar desalination and reinforces the extent to which estimates of annual productivity should consider variations in seasons.

**Fig. 20:**
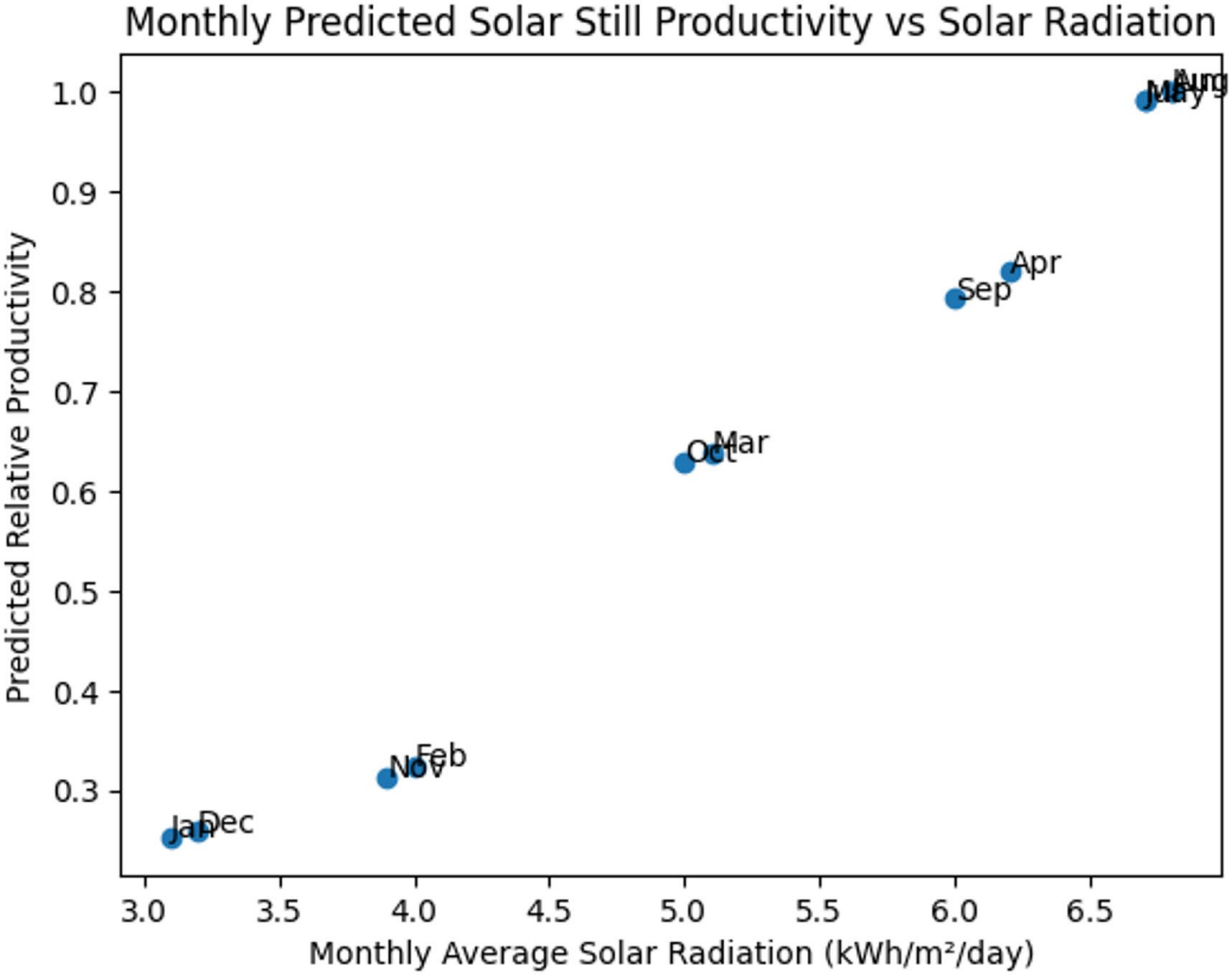
Monthly predicted productivity of the PCM-assisted solar still as a function of solar radiation considering seasonal PCM effectiveness.

The graph depicted in Fig. 20 shows the correlation between average monthly solar radiation and the predicted relative productivity of PCM-supported solar stills. The temperature parameterization of the phase change material accounted for variation in solar radiation and seasonality to estimate predicted productivity. The results show that the productivity increases and is almost directly proportional to solar radiation during months reporting higher levels of radiation and that a marked reduction occurs during winter due to both diminished input from solar irradiance and decreased melting of phase change material (PCM).

### Expected annual productivity taking into account seasonal PCM behavior

In addition to the seasonal change in the received solar radiation, annual productivity estimation should also consider the thermal performance of the phase change material (PCM) applied to the system. The PCM selected in this study has a melting temperature of 48 °C, also falling within the working temperature ranges commonly achieved in solar still basins under moderate to high radiation conditions.

During summer conditions, because of extremely high solar radiation intensity, basin water temperature often exceeds PCM melting temperature. The PCM completely melts in this binary state, and a large amount of latent heat is stored during its daytime operation. The heat collected during the day lingers and dissipates in the evenings and at night, providing additional warmth to the Basin and increasing freshwater production.

In winter, decreased solar radiation intensity and lower ambient temperature may limit the basin temperature from reaching the PCM melting point. If the PCM didn’t completely melt, it effectively comprises sensible heat storage instead of a latent heat storage medium, and that lowers its mass flow contribution in enhancing productivity.

The melting temperature of the selected phase change material (PCM) used in this study is 48 °C, which is within the normal working temperature range of solar still basins under moderate to high solar radiation conditions. The range of reported experimental basin water temperatures is between 45 °C and 70 °C, varying with solar radiation intensity and environmental conditions^34,35^. This figure implies complete PCM melting in the summer and high-radiation periods.

However, in winter months the diminished solar radiation and low ambient temperatures may prevent heat accumulation in the basin from reaching the PCM melting point. In this context, phase-change material (PCM) may start to partially melt, and its contribution switches from latent heat storage to sensible heat storage, which lowers the effectiveness of PCM aggregated in enhancing nighttime evaporation and freshwater productivity.

Monthly freshwater productivity can be estimated using the following relation to incorporate these seasonal effects into the formulation:

11 $$\:{P}_{i}={P}_{summer}\times\:\:\left(\frac{{I}_{i}}{{I}_{summer}}\right)\:\times\:\:{\eta\:}_{PCM,i}$$

Where.

$\:{P}_{i}$= estimated freshwater productivity during month $\:i$(kg.m⁻² day⁻¹).

$\:{\eta\:}_{PCM,i}$= seasonal PCM effectiveness factor.

The PCM effectiveness factor expresses how much the PCM participates in latent heat storage under varying climatic conditions. Inferred from existing observations documented in the literature, an ideal seasonal PCM effectiveness factor can be expressed approximately as:

- Summer months: $\:{\eta\:}_{PCM}\approx\:1.0$ (complete melting and full latent heat utilization).
- Spring and autumn months: $\:{\eta\:}_{PCM}\approx\:0.8\:\mathrm{\--}\text{}0.9$ (partial melting and moderate thermal contribution).
- Winter months: $\:{\eta\:}_{PCM}\approx\:0.5\:\mathrm{\--}\text{}0.6$ (limited melting; PCM mainly behaves as sensible heat storage).

The annual freshwater productivity of the system can then be estimated using the weighted average approach:

12 $$\:{P}_{annual}\text{}=\frac{\sum\:_{i=1}^{12}({P}_{i}\:\times\:{\:D}_{i})}{365}\:\text{}$$

Where.

$\:{P}_{annual}$= estimated annual productivity (kg.m⁻² day⁻¹).

$\:{P}_{i}$= estimated productivity for month $\:i$.

$\:{D}_{i}$= number of days in month $\:i$.

Based on the seasonal variation in solar radiation and accordingly reduced PCM effectiveness in winter months, PCM-aided solar still annual average productivity would be around 65–72% of peak summer productivity measured during experimental period. This estimate forms a more accurate foundation for the later economic and environmental valuations of the system.

### Economic analysis

The basic objective in all solar still systems is to minimize the cost per liter (CPL) of distillate water produced. The economic evaluation of the examined situations is performed using the principles presented in^14,23^. The first annualized cost (FAC) of the solar distillation system is given by

13 $$\:\mathrm{F}\mathrm{A}\mathrm{C}\:=\:\mathrm{C}\mathrm{R}\mathrm{F}\:\times\:\:\mathrm{P}\:$$

where P is the capital cost of the solar still, and CRF represents the capital recovery factor.

The formula for the Capital Recovery Factor (CRF) is expressed as:

14 $$\:\mathrm{C}\mathrm{R}\mathrm{F}=\frac{{i(1+i)}^{n}}{{(1+i)}^{n}\:-\:1}$$

where n is the number of years that the solar still will be used, assumed to be ten years, and i is the annual interest rate, the ASV of the solar distillation unit is given.

15 $$\:\mathrm{A}\mathrm{S}\mathrm{V}\:=\:\mathrm{S}\mathrm{S}\mathrm{F}\:\times\:\:\mathrm{S}$$

where SSF is the solar still’s salvage and S a system sinking fund factor (SFF). The source of S is:

16 $$\:\mathrm{S}\:=\:0.2\:\times\:\:\mathrm{P}$$

SSF is given by:

17 $$\:\mathrm{S}\mathrm{S}\mathrm{F}=\frac{i}{{(1+i)}^{n}\:-\:1}$$

The annual maintenance cost (AMC) is 15% of initial annual cost

18 $$\:\mathrm{A}\mathrm{M}\mathrm{C}\:=\:0.15\:\times\:\:\mathrm{F}\mathrm{A}\mathrm{C}\:$$

The total annual cost of the solar still is given below:

19 $$\:\mathrm{A}\mathrm{C}\:=\:\mathrm{F}\mathrm{A}\mathrm{C}\:+\:\mathrm{A}\mathrm{M}\mathrm{C}\:-\:\mathrm{A}\mathrm{S}\mathrm{V}$$

Finally, yields the cost per liter (CPL) of produced the freshwater yield,

20 $$\:\mathrm{C}\mathrm{P}\mathrm{L}=\frac{\mathrm{A}\mathrm{C}}{{\mathrm{P}}_{\mathrm{n}}}$$

Where P_n_ is the annual average production of distilled water.

**Fig. 21:**
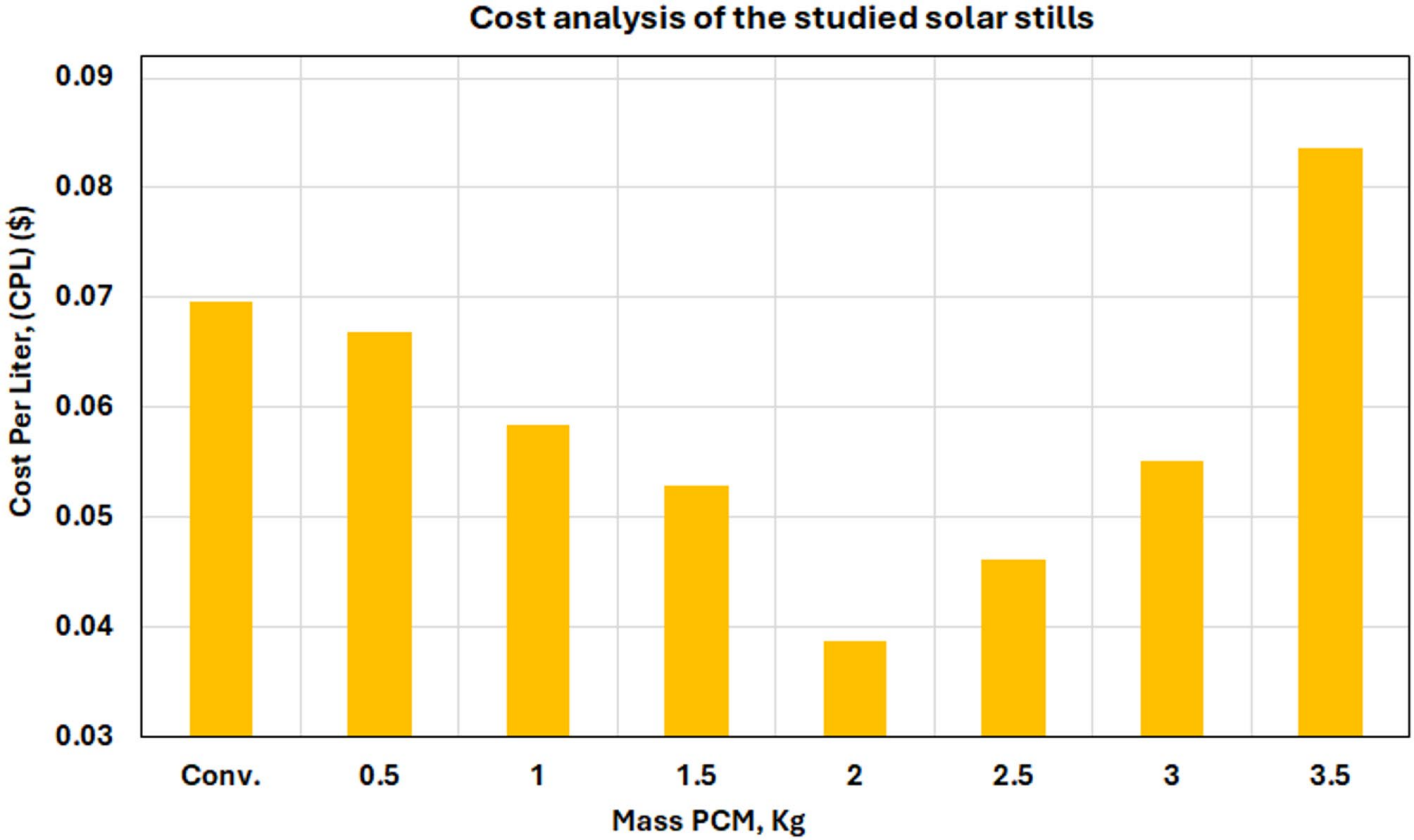
Variation of freshwater production cost per liter (CPL) for the conventional solar still and PCM-assisted configurations with different PCM masses.

Figure 21 shows a full economic analysis of all the configurations that were tried. This sets cost-effectiveness standards that are important for putting the ideas into practice. The bar chart reveals that the regular solar still costs the most per liter at $0.068, while the best 2.5 kg PCM system costs $0.031 per liter, which is a 54.4% savings. This economic advantage is due to the fact that the greater productivity rates of freshwater outweigh the higher costs of PCM materials. The economic study shows that PCM-enhanced solar stills are a good business idea. It shows that putting more money into PCM materials leads to big savings on operating costs since they work better.

### Environment impact analysis

The NCEM (net carbon dioxide mitigation) in tons $\:{\mathrm{C}\mathrm{O}}_{2}$^6^,.

21 $$\:\mathrm{N}\mathrm{C}\mathrm{E}\mathrm{M}=\frac{1.58\:\left[\left({\mathrm{E}}_{\mathrm{o}\mathrm{u}\mathrm{t}\:}\times\:\:\mathrm{n}\right)-{\mathrm{E}}_{\mathrm{i}\mathrm{n}}\right]}{1000}\:\:,\left(\mathrm{t}\mathrm{o}\mathrm{n}\mathrm{s}\right)$$

At the time, $$\:{\mathrm{C}\mathrm{O}}_{2}$$ is being valued at around $14 per ton. As a result, the carbon credit that is secured is given by:

22 $$\:\mathrm{C}\mathrm{C}\mathrm{G}=\mathrm{N}\mathrm{C}\mathrm{E}\mathrm{M}\:\times\:\:\mathrm{c}\mathrm{o}\mathrm{s}\mathrm{t}\:\mathrm{o}\mathrm{f}\:{\mathrm{C}\mathrm{O}}_{2},\:\mathrm{t}\mathrm{r}\mathrm{a}\mathrm{d}\mathrm{e}\mathrm{d}\:\mathrm{p}\mathrm{e}\mathrm{r}\:\mathrm{t}\mathrm{o}\mathrm{n}.$$

**Fig. 22:**
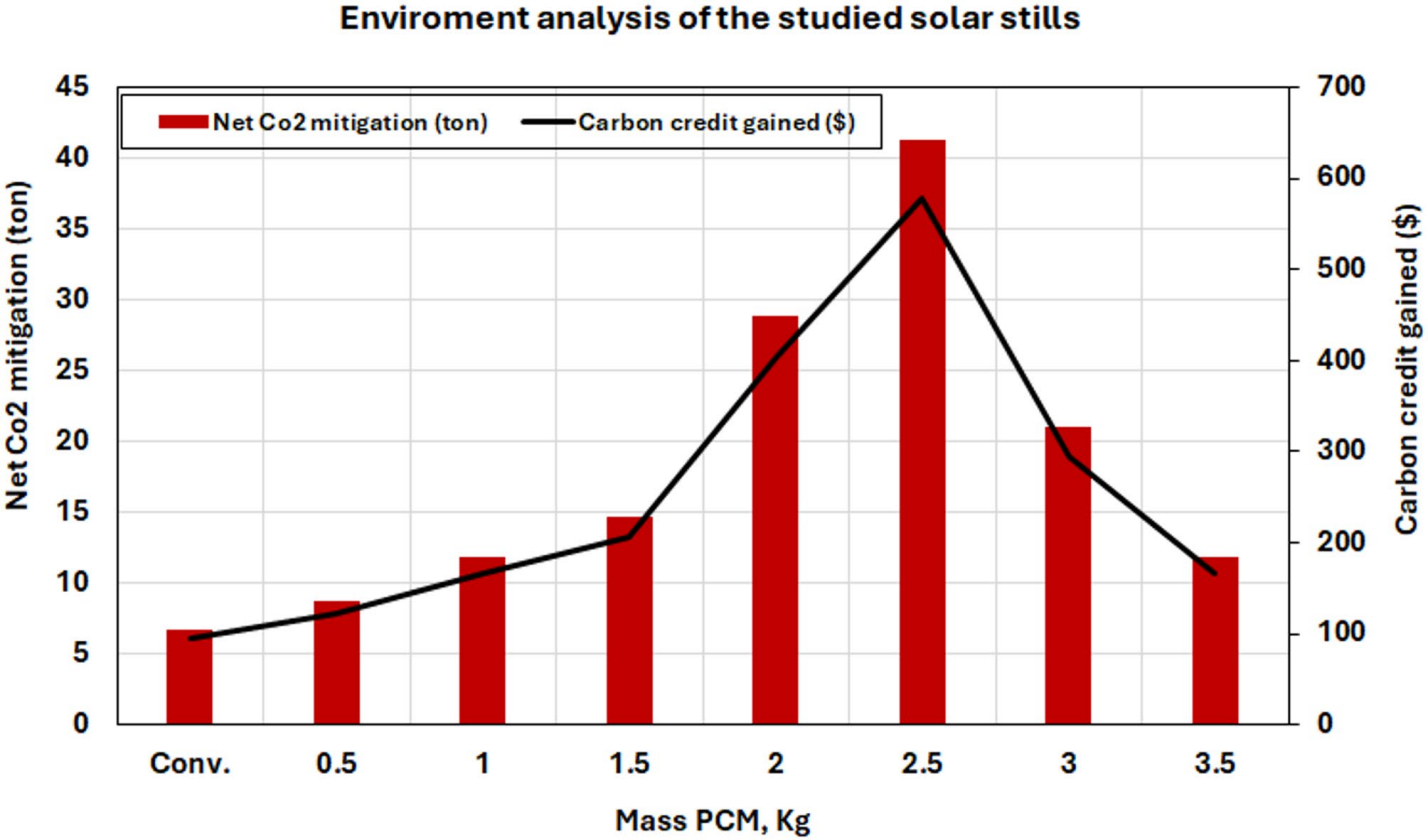
Net CO_2_ product mitigation and credit of carbon gained for the studied cases.

Figure 22 estimates the ecological advantages of PCM-enhanced solar distillation systems via a carbon dioxide mitigation analysis. The data indicates that the ideal 2.5 kg PCM system realizes a CO₂ reduction of 41.26 tons over its 10-year operational lifespan, yielding $577.61 in carbon credits at prevailing market pricing ($14/ton $$\:{\mathrm{C}\mathrm{O}}_{2}$$). The environmental advantages, along with less reliance on fossil fuels for freshwater generation, illustrate the sustainability benefits of PCM-enhanced solar distillation technology.

To validate the improvements of the proposed PCM-assisted solar still configuration, researchers conducted a comprehensive comparison with previously reported works in the literature, as presented in Table 4. The comparison concentrates on four key performance parameters considered in this study, such as energy efficiency, exergy efficiency, economic price (cost per liter), and environmental impact. The obtained results ascertain that the proposed PCM-assisted solar still showed superior thermodynamic and economic performance compared to the previously reported systems. There was an improvement of approximately 43.2% in energy efficiency compared to the previous study, reaching from 17.3 to 24.8. Similarly, exergy efficiency increased from 4.9% to 7.1%, representing an enhancement of almost 44.9%. The cost of freshwater production per liter decreased from $0.049 to $0.031, with a decrease of about 58.1%.⁶ Economically, the developing system is more commonly used as it highlights its economic viability due to its lower operating costs associated with land area than any other device. In addition, the environmental assessment found that CO₂ mitigation increased from 34.74 tons to 41.26 tons during the system service life compared with traditional use, an increase of nearly 18.8%. The data shows that the best approach for PCM incorporation applied in this research has achieved better thermal storage capacity and higher freshwater output while enhancing global sustainability performance over configurations reported earlier.

**Table 4:** Comparative 4E performance analysis between the present study and previously published work.

Cases	Present work		Pervious work^36^		Effective %
	Conventional SS	Modified SS with 2.5 kg of 48 SP	Conventional SS	Modified SS 2.5 kg of 42 SP	
Energy analysis	10.4%	24.8%	11.6%	17.3%	43.2%$\:\uparrow\:$
Exergy analysis	1.5%	7.1%	2.8%	4.9%	44.9%$\:\uparrow\:$
Economic Analysis (CPL)	0.068$	0.031$	0.059$	0.049 $	58.1%$$\:\downarrow\:$$
Net CO_2_ Mitigation	7.24 ton	41.26 ton	14.93 ton	34.74 ton	18.8%$\:\uparrow\:$

### Hybrid solar still system performance

#### Enhanced thermal characteristics with hot domestic water integration

**Fig. 23:**
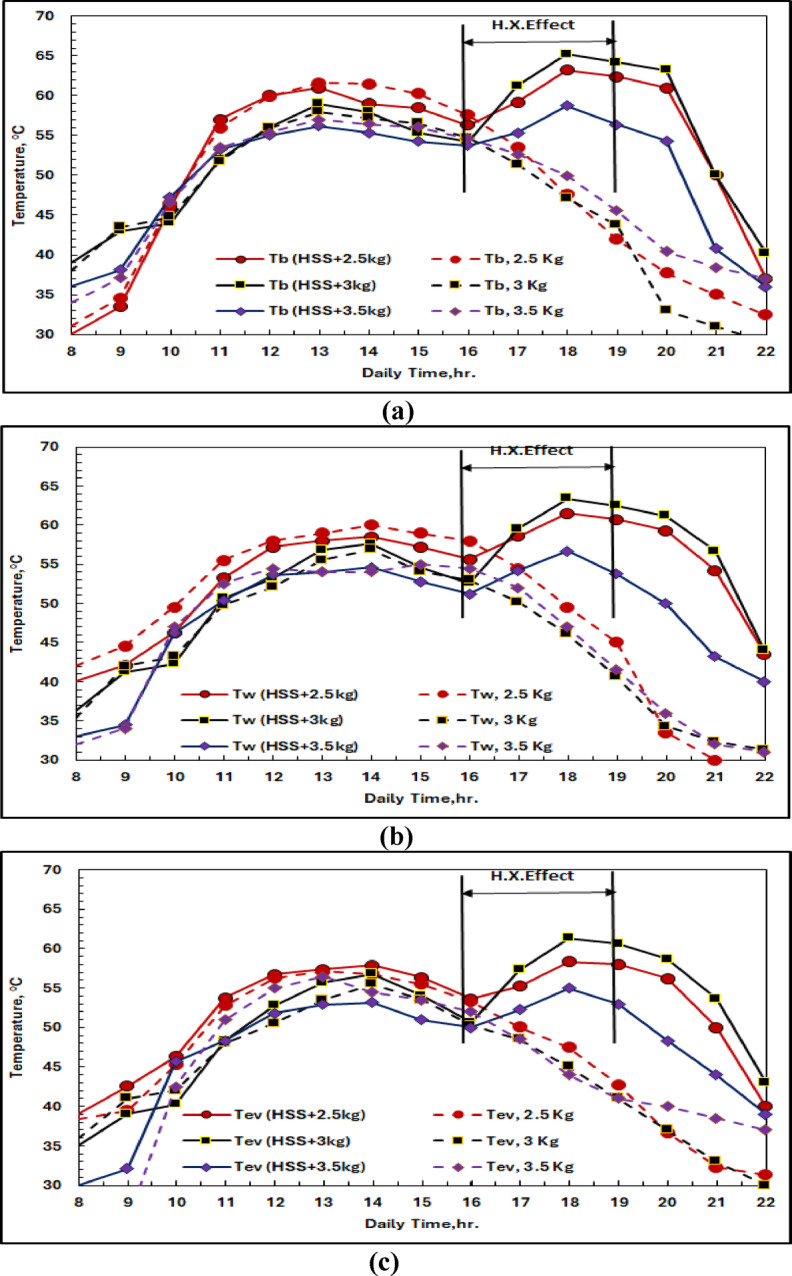
The Effect of HDW Tank on HSS system with 2.5, 3, and 3.5 kg of SP48 according to (**a**) Basin Temperature, (**b**) Saline Water Temperature, and (**c**) Evaporation Temperature.

Figures 23 (a-c) demonstrate that the hot domestic water (HDW) exhibits improved thermal performance with varying masses of phase change materials (PCMs), which are optimized for different temperature and PCM masses (2.5, 3, and 3.5 kg). The plots indicate substantial temperature enhancements, with the 3 kg PCM + HDW system reaching a peak basin temperature of 65.24 °C. Hot water (average temperature: 59 °C) flows rate at75.5 ml/min to the heat exchanger network in the HDW integration system when solar radiation intensity decreases to 716 W/m² from time 16:00.

The temperature rises of the basin are 61.23 °C, 65.24 °C, and 64.20 °C for PCM masses of 2.5 kg, 3 kg, and 3.5 kg, respectively, during a three-hour-long HDW operational time, as shown in Fig. 22 (a). Figure 23 (b) shows improvements in saltwater temperature of 28.08%, 37.5%, and 34.7% for these configurations as compared to the non-HDW cases. Figure 22 (c) shows the temperature rise of water vapor by 16.4 °C (36.3% enhancement) in the HDW coupling, which contributes to better heat transfer during evaporation actions.

#### PCM thermal dynamics in hybrid configuration

**Fig. 24:**
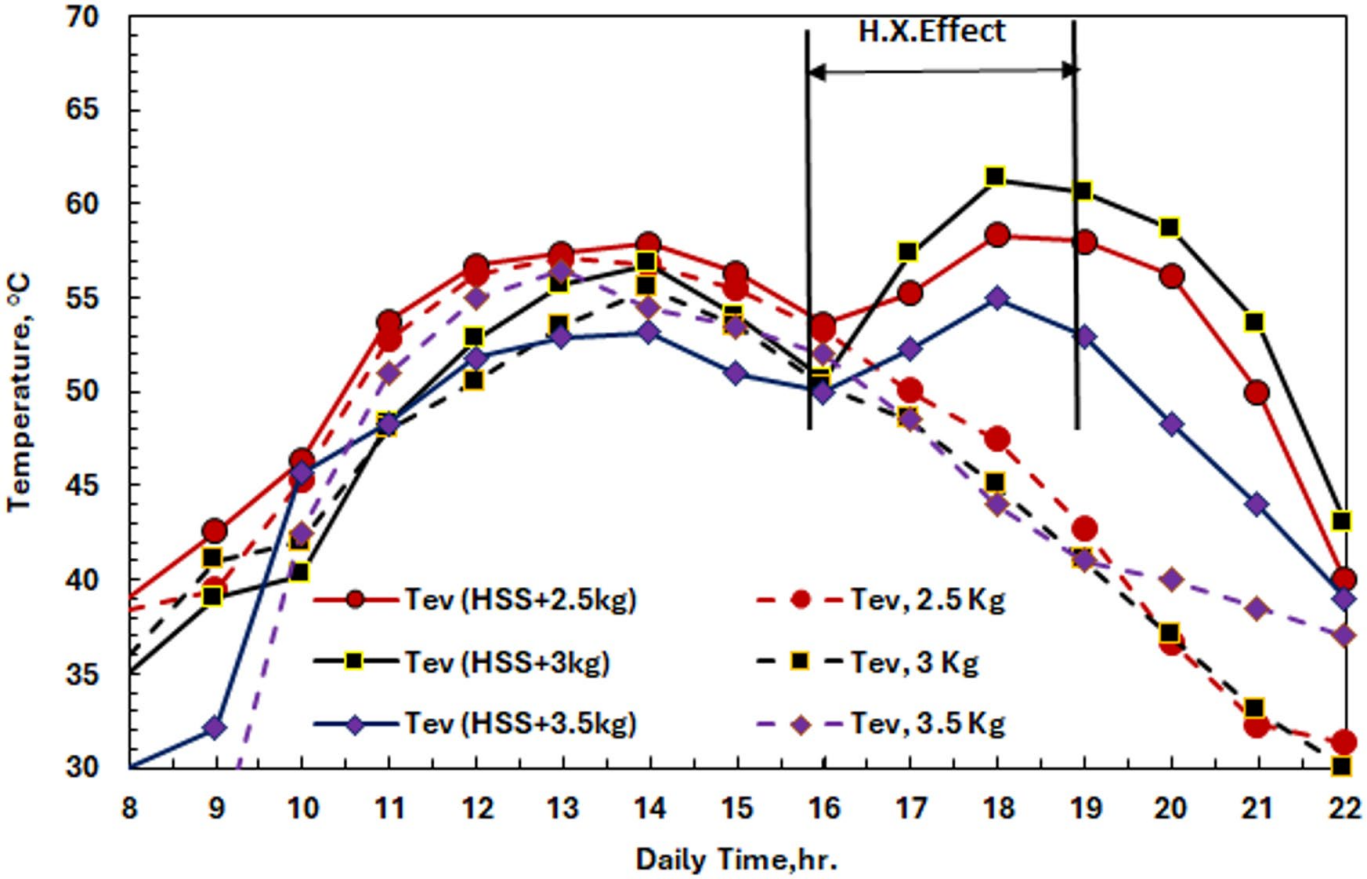
The Effect of HDW on 2.5, 3 and 3.5 kg Masses of the PCM Temperatures.

Figure 24 shows the positive effect of HDW integration on PCM thermal behavior. It can be seen that for all PCM putty masses, the melting point of 48 °C was obtained, but the integration of HDW increased the liquefaction phase time, indicating more input energy into the exchanger incorporated in PCM from both solar radiation and hot water stored. The longer liquid phase facilitates further heat input from the basin to the water, improving system performance during long cloudy periods with low solar radiation.

#### Hybrid System Productivity Performance

**Fig. 25:**
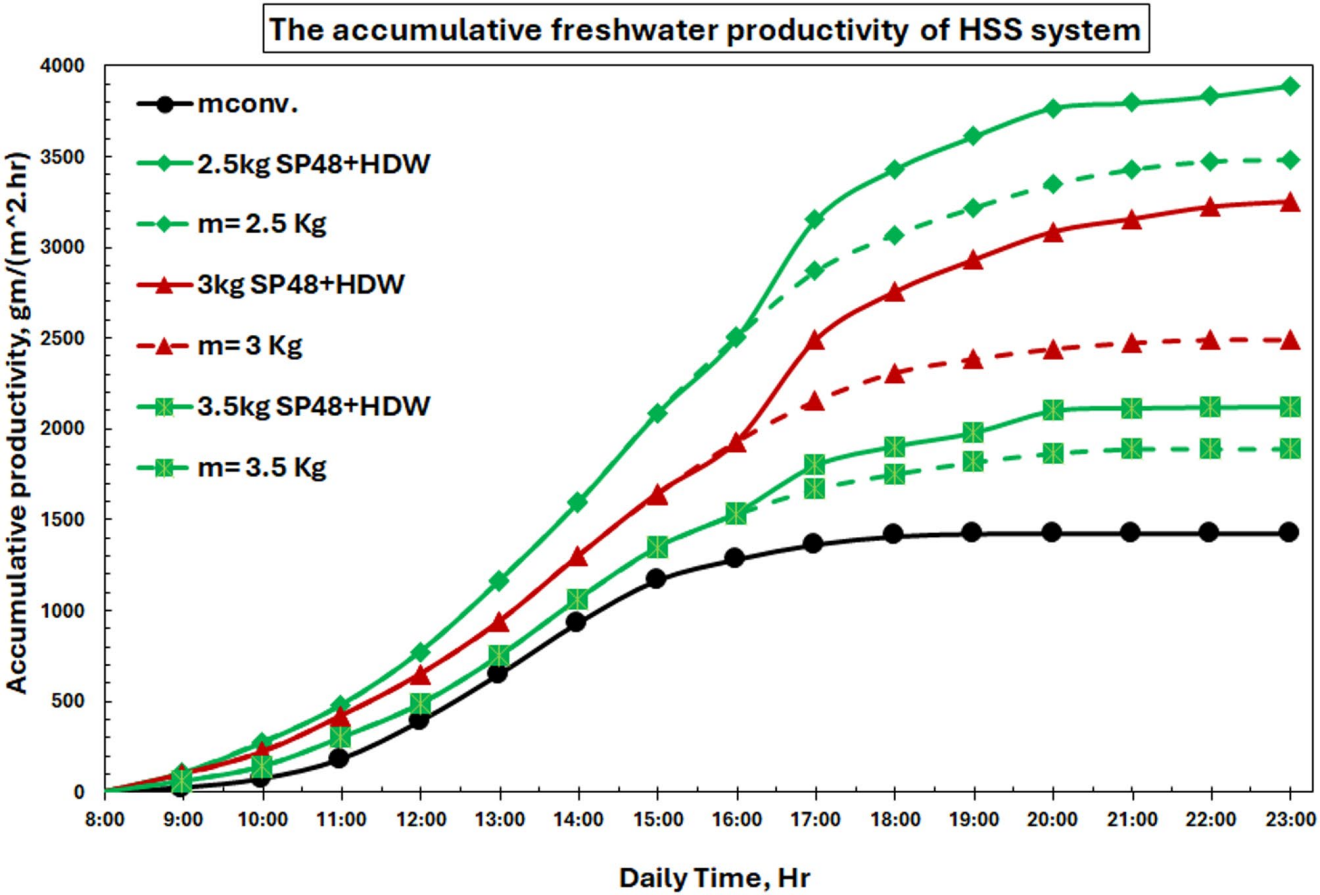
Cumulative fresh water productivity of HSS at 2.5,3, and 3.5 kg SP48 with/without HDW.

Figure 25 shows the cumulative freshwater productivity for hybrid solar still systems, which offer significant performance improvements compared to traditional and PCM-only models. Until 16:00, the productivity of hybrid systems shows limited variance; after this time, the integration of HDW resulted in significant efficiency gains. Compared with the non-HDW systems, for PCM masses of 2.5 kg and 3 kg, the freshwater productivity was achieved as follows: 28.5%, 53.3%, and 16.2%.

**Fig. 26:**
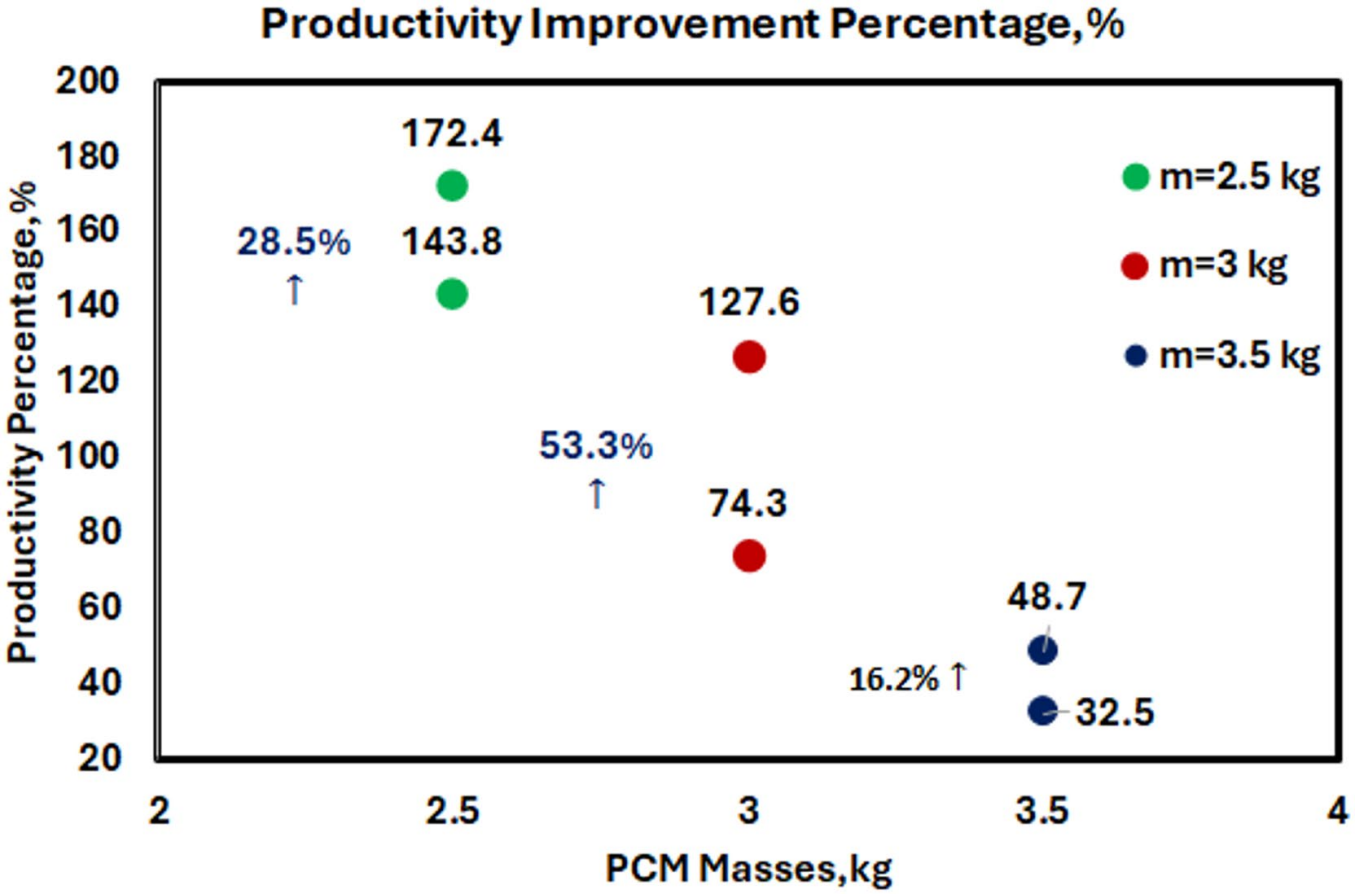
The improvement percentage of freshwater productivity in HSS system.

Figure 26 quantifies the percentages of productivity improvements, with the 3 kg PCM + HDW being the best for hybrid operation, where it recorded a 53.3% production improvement. This optimal mass is higher than the independent PCM system (2.5 kg) because of the extra thermal energy contributing from HDW, which allows more PCM to be fully used.

#### Economic analysis of hybrid systems

The economic evaluation in Tables 5 and 6 shows that the 3 kg PCM modified system costs $0.044 per liter, whereas the hybrid system costs $0.053 and the conventional systems cost $0.068. The hybrid system costs 65.7% more to set up ($235.8 compared to $142.3), but it increases productivity by 127.5%, which lowers costs by 28.3% compared to traditional systems. The economics of the modified PCM system are best, with cost falling by 54.5% and capital increasing by only 4.6%.

In this economic evaluation of the current hybrid system, hot domestic water supplied to the system was considered an available thermal input, and its direct heating cost was not included separately in the cost estimation. As such, the reported hybrid-system economic results present a best-case comparative scenario for assessing the effect of additional thermal input on system productivity. If an outside energy resource were used to heat the domestic water, of course, the total operational cost would increase in conjunction with the hybrid system. This assumption is relevant when interpreting the cost-effectiveness of the HSS configuration.

**Table 5:** Cost analysis of the studied solar still systems.

Item	*P* ($)	FAC ($)	ASV ($)	AMC ($)	AC ($)	Pd (L/day)	Pn (L/year)	CPL ($)
CSS	142.3	31.78	1.21	4.76	35.3	1.43	522	0.068
Modified SS at 3 kg SP48	148.8	35.56	1.16	5.33	39.7	2.49	909	0.044
Hybrid SS at 3 kg SP48 with HDW	235.8	56.36	1.84	8.45	62.9	3.25	1186.25	0.053

**Table 6:** Rate of the analysis of cost per studied solar still cases.

Item	Capital Cost Increment (%)	Rate of Freshwater Productivity (%)	Improvement Rate of CPL (%)
CSS	0	0	0
Modified SS at 3 kg SP48	4.6%	74.2	54.5
Hybrid SS at 3 kg SP48 with HDW	65.7%	↑127.5	↓28.3
Rate of change	$\:\varDelta\:\:61.4$	$\:\varDelta\:\:$53.2	$\:\varDelta\:\:26.2$

However, it is important to note that the forecasting mechanism applied in this cost analysis is not based on PCM performance degradation or periodic replacement over time; thus, we assume stable PCM performance over the duration of interest. Thus, the financial indicators reported should be considered comparative estimates under the assumptions chosen. This will require an important amount of validation over an extended period of time, wherein the real-life long-term cost, energy use, and emissions can be optimized in PCM, same as hybrid configurations.

This comprehensive investigation shows that solar stills with PCM improve thermal performance, freshwater productivity, energy efficiency, economic viability, and environmental sustainability a lot compared to regular systems. The independent 2.5 kg PCM configuration is found to be the optimal single-system design, and the combined 3 kg PCM + HDW configuration is determined as the optimal hybrid system.

## Conclusions

Incorporating PCMs with solar still systems would significantly improve freshwater productivity in Egypt, based on this extensive scientific study’s results. Here are the main results:

- The PCM-based 2.5 kg system obtained 73.8% more fresh water/day (2482 gm/m²/day over 1428), and it demonstrated the highest energy efficiency (24.77%) as well as exergy efficiency (7.10%).
- The basin water temperature (59 °C) was significantly increased compared with that of conventional systems on-site (53 °C), and the operation time was prolonged into night periods by utilizing PCM thermal storage; this result led to a notably better thermal performance.
- With a 54.4% lower cost (£0.031/L vs. £0.068 for the conventional system), the economic viability of the system was verified, and a short payback period also elucidated that the technology was commercially practicable.
- The environmental benefits equal 41.26 tons of CO₂ reduction over 10 years with a value of $577.61, contributing to the fight against climate change.
- For stand-alone systems, 2.5 kg of PCM is the best amount, as confirmed by a parametric study. For 3 kg of PCM and HDW systems, the performance is increased by 53.3%. SP. 48 PCM, having a melting point at 48 °C, was found to be the most appropriate for hot, dry climates, considering full use of phase transition can be achieved at proper mass loadings.
- A comprehensive 4E analysis method, including energy, exergy, economics, and environment, was presented together with a robust 5-day testing schedule that ensures reliable experimental validation.
- HDW has successfully applied its temperature management methodology for hybrid systems, which has set a new standard of performance and productivity for solar stills.
- PCM materials are commercially available; therefore, the supply chain can be guaranteed for deployment in low-tech areas.

Technological breakthroughs indicate that there are fast-developing sustainable desalination technologies that could be implemented everywhere, above all in North Africa, where water is so scarce, and in the Middle East. The results of current research show that PCM assisted with hybrid solar still systems can be considered effective and sustainable approaches for increasing freshwater production in hot climates. But wider practical deployment may rely on further long-term validation studies, seasonal performance evaluation, and economic modeling under true operating conditions.

## Limitations and future work

Despite the significant performance improvement when a medium-temperature PCM was applied to the solar still, several limitations should be acknowledged. These limitations do not invalidate the current findings but provide clear avenues for enhancement in subsequent studies:

- The experiments were conducted during the summer season on the 10th of Ramadan city in Egypt. These might be a feature of high-irradiation environments, but do not fully account for seasonal and geographic variation. The generalizability of these results, or season dependent PCM selections according to local climates, needs to be confirmed by more extensive theoretical and experimental work in different locations over several seasons.
- The study is limited to a fixed basin size for a small amount of PCM, also ensuring it is thermally predictable; hence, larger systems need to be considered. Future studies should include testing other solar-still variations. In addition, long-term aspects of performance, such as material degradation, maintenance requirements, and recyclability for both the PCM and structural components, should be studied comprehensively to enable realistic deployment.
- More comprehensive techno-economic analyses that incorporate realistic commercial-scale cost data are needed to assess financial feasibility across a range of market scenarios, consistent with guiding the technology’s advancement toward higher TRLs.
- Despite produced water in our study meets WHO drinking-water standards, long-term operation and large-scale applications should constantly monitor the water quality.

## Data Availability

The datasets used and/or analyzed during the current study are available from the corresponding author on reasonable request.

## List of symbols

A: Basin area (m^2^)
I: The solar radiation intensity (W/m^2^)
P: Capital cost ($)
T: The temperature (°C)
η: efficiency (%)

## Subscript

B: Basin water
en: Energy
ex: Exergy
ev: Water vapor (evaporation)
in: Input
u: Useful
G: Glass temperature
W: Salty water

## Abbreviations

AC: Annual cost
AM: Ambient measurement
CPL: Cost per liter of produced water
CRF: Capital recovery factor
CSS: Conventional solar still
HDW: Hot domestic water
HSS: Hybrid solar still
PCM: Phase change material
PCM-SS: PCM-assisted solar still
4E: Energy, Exergy, Economic, Environmental
SD: Solar distillation
TDS: Total dissolved solids
TES: Thermal energy storage

## References

[1] Jafaripour, M., Roghabadi, F. A., Soleimanpour, S. & Sadrameli, S. M. Barriers to implementation of phase change materials within solar desalination units: Exergy, thermal conductivity, economic, and environmental aspects review. *Desalination* **546**, 116191. 10.1016/j.desal.2022.116191 (2023).

[2] El-Ghandour, M., Elminshawy, N. A. S. & Soliman, M. S. Performance of a solar still combined with external energy storage and Fresnel lens concentrator. *Journal of Energy Storage* **128**, 117222. 10.1016/j.est.2025.117222 (2025).

[3] Dhivagar, R., Omara, A. A. M., Kannan, K. G., Prabakaran, R. & Kim, S. C. Dual-Organic Phase Change Material: A Sustainability-Oriented Approach for High-Performance Solar Stills. *International Journal of Energy Research* **2025**, 1. 10.1155/er/6182051 (2025).

[4] Dhivagar, R. et al. Sustainable solar still desalination using beeswax and paraffin wax phase change materials: A 5E comparison toward emerging efficient systems. *Thermal Science and Engineering Progress*10.1016/j.tsep.2025.103994 (2025).

[5] Moreno, S., Álvarez, C., Hinojosa, J. F. & Maytorena, V. M. Numerical analysis of a solar still with phase change material under the basin. *J. Energy Storage*10.1016/j.est.2022.105427 (2022).

[6] Bilal, A., Jamil, B., Haque, N. U. & Ansari, M. A. Investigating the effect of pumice stones sensible heat storage on the performance of a solar still. *Groundwater for Sustainable Development* 100228. 10.1016/j.gsd.2019.100228 (2019).

[7] Hansen, R. S. et al. Utilization of PCM in inclined and single basin solar stills to improve the daily productivity. *Materials Today: Proceedings* **62**, 967–972. 10.1016/j.matpr.2022.04.092 (2022).

[8] Tiwari, S. & Rathore, K. S. Performance enhancement of solar still for water desalination integrated with thermal energy storage. *Mater. Today Proc.* **74**, 202–206. 10.1016/j.matpr.2022.08.048 (2021).

[9] Ajdari, H. & Ameri, A. Performance assessment of an inclined stepped solar still integrated with PCM and CuO/GO nanocomposite as a nanofluid. *J. Build. Eng.* **49**, 104090. 10.1016/j.jobe.2022.104090 (2022).

[10] Elgendi, M., Kabeel, A. E. & Essa, F. A. Improving the solar still productivity using thermoelectric materials: A review. *Alexandria Eng. J.* **65**, 963–982. 10.1016/j.aej.2022.10.011 (2023).

[11] Soliman, M. S., Yassen, Y. E. S., El-Nahhas, K. & Elminshawy, N. A. S. Performance investigation of solar still utilizing affordable thermal storage material and parabolic trough concentrator: An experimental investigation. *Process Saf. Environ. Prot.*10.1016/j.psep.2025.107473 (2025).

[12] Bady, M., Attia, M. E. H., Kabeel, A. E., Elminshawy, N. A. S. & Sathyamurthy, R. Synergizing Water Desalination of A Conical Solar Distiller Using Copper Fins Filled with Phosphate As A Porous Sensible Heat Storage Material. *Int. J. Thermophys.*10.1007/s10765-025-03586-6 (2025).

[13] Elbar, A. R. A., Yousef, M. S. & Hassan, H. Energy, exergy, exergoeconomic and enviroeconomic (4E) evaluation of a new integration of solar still with photovoltaic panel. *J. Clean. Prod.* **233**, 665–680. 10.1016/j.jclepro.2019.06.111 (2019).

[14] Yousef, M. S. & Hassan, H. Energetic and exergetic performance assessment of the inclusion of phase change materials (PCM) in a solar distillation system. *Energy Conversion and Management* **179**, 349–361. 10.1016/j.enconman.2018.10.078 (2019).

[15] Dhivagar, R., Suraparaju,Jidhesh, S. K. & Kim, S. C. Integration of solar photovoltaic panel and A46 phase change material in double-slope solar still: A progressive approach for performance enhancement. *Sep Purif. Technol. 374 March*. 10.1016/j.seppur.2025.133754 (2025).

[16] Suraparaju, S. K. & Natarajan, S. K. Effect of natural sisal fibre on enhancing the condensation rate of solar still for sustainable clean water production. *Thermal Science and Engineering Progress* **36**, 101527. 10.1016/j.tsep.2022.101527 (2022).

[17] Raju, V. R. & Narayana, R. L. Effect of flat plate collectors in series on performance of active solar still for Indian coastal climatic condition. *J. King Saud Univ. Eng. Sci.* **30**(1), 78–85. 10.1016/j.jksues.2015.12.008 (2018).

[18] Abdullah, A. S. et al. Enhancing trays solar still performance using wick finned absorber, nano- enhanced PCM. *Alexandria Eng. J.* **61**(12), 12417–12430. 10.1016/j.aej.2022.06.033 (2022).

[19] Manokar, A. M. et al. Comparative study of an inclined solar panel basin solar still in passive and active mode. *Sol Energy*. **169**, 206–216. 10.1016/j.solener.2018.04.060 (2018).

[20] Kabeel, A. E., Abdelgaied, M. & Eisa, A. Enhancing the performance of single basin solar still using high thermal conductivity sensible storage materials. *J. Clean. Prod.* **183**, 20–25. 10.1016/j.jclepro.2018.02.144 (2018).

[21] Suraparaju, S. K. et al. Energy, exergy, economic and environmental (4E) analyses of solar still with paraffin wax as phase change energy storage material. *Mater. Today Proc.* **90**(xxxx), 1–5. 10.1016/j.matpr.2023.03.345 (2023).

[22] Ahmed, H., Najib, A., Zaidi, A. A., Naseer, M. N. & Kim, B. Modeling, design optimization and field testing of a solar still with corrugated absorber plate and phase change material for Karachi weather conditions. *Energy Rep.* **8**, 11530–11546. 10.1016/j.egyr.2022.08.276 (2022).

[23] Rashidi, S., Rahbar, N., Valipour, M. S. & Esfahani, J. A. Enhancement of solar still by reticular porous media: Experimental investigation with exergy and economic analysis. *Appl. Therm. Eng.* **130**, 1341–1348. 10.1016/j.applthermaleng.2017.11.089 (2018).

[24] Siddula, S. et al. Triangular and single slope solar stills: Performance and yield studies with different water mass. *Energy Rep.* **8**, 480–488. 10.1016/j.egyr.2022.10.225 (2022).

[25] Shah, R., Makwana, M., Makwana, N. & Desai, R. Performance analysis of black gravel solar still. *Mater. Today Proc.* **72**, 1000–1006. 10.1016/j.matpr.2022.09.115 (2023).

[26] Mohamed, A. F., Hegazi, A. A., Sultan, G. I. & El-Said, E. M. S. Augmented heat and mass transfer effect on performance of a solar still using porous absorber: Experimental investigation and exergetic analysis. *Appl. Therm. Eng.* **150**, 1206–1215. 10.1016/j.applthermaleng.2019.01.070 (2019).

[27] Kabeel, A. E., Abdelaziz, G. B. & El-Said, E. M. S. Experimental investigation of a solar still with composite material heat storage: Energy, exergy and economic analysis. *J. Cleaner Prod.* **231**, 21–34. 10.1016/j.jclepro.2019.05.200 (2019).

[28] Singh, H. N. & Tiwari, G. N. Monthly performance of passive and active solar stills for different Indian climatic conditions. *Desalination* **168**(1–3), 145–150. 10.1016/j.desal.2004.06.180 (2004).

[29] Hamadou, O. A. & Abdellatif, K. Modeling an active solar still for sea water desalination process optimization. *Desalination* **354**, 1–8. 10.1016/j.desal.2014.09.019 (2014).

[30] Manokar, A. M. et al. Effect of mass flow rate on fresh water improvement from inclined pv panel basin solar still. *Mater. Today Proc.* **32** (xxxx), 374–378. 10.1016/j.matpr.2020.02.051 (2019).

[31] Dhivagar, R. A Concise Review on Productivity and Economic Analysis of Auxiliary-Component-Assisted Solar Stills. *Energy Technology* **9**(11), 1–12. 10.1002/ente.202100501 (2021).

[32] Dhivagar,Jidhesh, R., Kannan, K. G. & Kim, S. C. Thermo-economic and environmental assessment of a sustainable Trigeneration double slope solar still integrated with a single-side mounted photovoltaic panel and electrical heating element. *Sep. Purif. Technol.* **380,P1** (135226). 10.1016/j.seppur.2025.135226 (2026).

[33] Moffat, R. J. Using uncertainty analysis in the planning of an experiment. *J. Fluids Eng.* **107**(2), 173–178. 10.1115/1.3242452 (1985).

[34] Kabeel, A. E. & Abdelgaied, M. Improving the performance of solar still by using PCM as a thermal storage medium under Egyptian conditions. *Desalination* **383**, 22–28. 10.1016/j.desal.2016.01.006 (2016).

[35] Shalaby, S. M., El-Bialy, E. & El-Sebaii, A. A. An experimental investigation of a v-corrugated absorber single-basin solar still using PCM. *Desalination* **398**, 247–255. 10.1016/j.desal.2016.07.042 (2016).

[36] Mustafa, M. S., Mousa, M. G., Dawood, M. M. K., Mansour, T. M. & Nabil, T. Energy, Exergy, Economic and Environmental Analysis of Single Slope Solar Still System using Phase Change Material. *Results Eng.* **24**, 103108. 10.1016/j.rineng.2024.103108 (2024).

